# Control of Crystallinity and Stereocomplexation of Synthetic Carbohydrate Polymers from d‐ and l‐Xylose

**DOI:** 10.1002/anie.202013562

**Published:** 2021-01-07

**Authors:** Thomas M. McGuire, Jessica Bowles, Edward Deane, Elliot H. E. Farrar, Matthew N. Grayson, Antoine Buchard

**Affiliations:** ^1^ Department of Chemistry University of Bath Centre for Sustainable and Circular Technologies Claverton Down Bath BA2 7AY UK

**Keywords:** carbohydrates, polyether, polysaccharide mimics, ring-opening polymerisation, stereocomplex, xylose

## Abstract

Manipulating the stereochemistry of polymers is a powerful method to alter their physical properties. Despite the chirality of monosaccharides, reports on the impact of stereochemistry in natural polysaccharides and synthetic carbohydrate polymers remain absent. Herein, we report the cocrystallisation of regio‐ and stereoregular polyethers derived from d‐ and l‐xylose, leading to enhanced thermal properties compared to the enantiopure polymers. To the best of our knowledge, this is the first example of a stereocomplex between carbohydrate polymers of opposite chirality. In contrast, atactic polymers obtained from a racemic mixture of monomers are amorphous. We also show that the polymer hydroxyl groups are amenable to post‐polymerisation functionalization. These strategies afford a family of carbohydrate polyethers, the physical and chemical properties of which can both be controlled, and which opens new possibilities for polysaccharide mimics in biomedical applications or as advanced materials.

## Graphical abstract

Synthetic carbohydrate polyethers, which are polysaccharide mimics, are synthesised by regioregular anionic ring‐opening polymerisation of oxetanes produced from d‐ or l‐xylose. By exploiting the chirality of xylose and varying stereochemical composition, the crystallinity of the resulting materials can be controlled. Mixing together homochiral polymers of opposite configuration results in a stereocomplex with enhanced thermal properties.

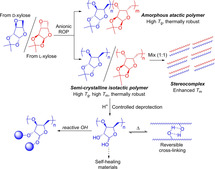

Carbohydrates represent a renewable resource with tremendous potential for synthetic polymers.^[1]^ In addition to the low cost and abundance of starting materials, their multiple hydroxyl groups offer significant functionalization potential and broadens the application scope of the resulting materials.^[2]^ Moreover, the chirality of monosaccharides presents an opportunity to further control the physical properties of sugar‐derived polymers. However, reports on the impact of chirality and tacticity, as well as attempts at stereocomplexation in carbohydrate polymers remain absent.^[3]^ In particular, stereocomplexation, the interaction between two complementary stereoregular polymers, has emerged as a powerful tool to improve the physical properties of materials, to delay release and biodegradation in the case of drug delivery systems, as well as to favour interactions between enantiomeric polymers and biomacromolecules.^[4]^

Amongst other techniques, Ring‐Opening Polymerisation (ROP) has been successfully employed to synthesize sugar‐based polymers which maintain their cyclic, furanose or pyranose, core, and as a result display high glass‐transition temperatures (*T*_g_). Polymers formed this way often feature carbonate[^2a^, ^2b^, ^2c^, ^2d^, ^2e^, ^2f^] or related (e.g. thiocarbonate^[5]^ or phosphoester^[6]^) linkages, which can have limited thermal stability and be susceptible to chemical (e.g. hydrolytic) degradation,^[2b]^ and may not be suited to some applications. The ROP of anhydrosugar derivatives can also enable access to polysaccharide mimics (carbohydrate polyethers).^[1e]^ Polyethers are generally more stable than their carbonyl‐containing analogues.^[7]^ Consequently, whilst they can exhibit biodegradability (e.g. PEG/PEO),^[8]^ polyethers also find applications in thermally active and non‐inert environments such as batteries (as solid electrolytes).^[9]^ However, controlled ROP of anhydrosugars can be challenging and systematic study of the resulting material properties has been limited compared to investigations into biomedical applications.

We,^[10]^ amongst others,^[11]^ have identified xylofuranose diols as promising precursors for the preparation of functionalized polymers, owing to their abundance and low cost. In particular, Uryu and co‐workers have previously reported the polymerization of anhydro‐functionalized xyloses,^[12]^ including the cationic polymerization of 3,5‐anhydroxylofuranose derivative, **D‐1**, under high vacuum in the presence of PF_5_ or BF_3_⋅OEt_2_.^[13]^ A highly regioregular, isopropylidene‐functionalized [3→5]‐xylan mimic (poly(**D‐1**)) was obtained, albeit with no demonstrable control over the degree of polymerization. Notably, efforts to polymerize **D‐1** with anionic initiators failed. Herein, we report the controlled anionic polymerization of **D/L‐1** and the impact of the polymer stereochemical composition, as well as of post‐polymerization modifications, on the material thermal properties. We also report the formation of a novel stereocomplex formed by mixing both homochiral polymers (Figure 1).

**Figure 1:**
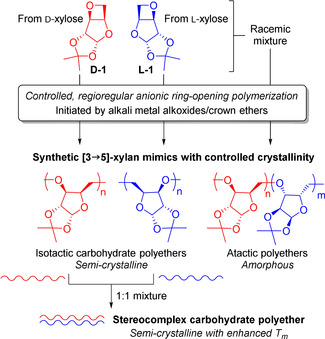
Anionic ring‐opening polymerization of xylose‐derived oxetane to enantiomerically pure, regioregular isotactic polymers or racemic atactic copolymers, and formation of semi‐crystalline stereocomplex.

1,2‐Acetalization of d‐xylose was performed using a modified literature procedure.^[14]^ Monotosylation of 1,2‐O‐isopropylidene‐α‐d‐xylofuranose followed by cyclisation delivered **D‐1** in quantitative yields (Scheme 1). Purification by distillation over CaH_2_ gave monomer of sufficient purity for polymerization. Notably, the synthesis of **D‐1** does not require column chromatography, and reactions were possible on a 20 g scale.

**Scheme 1:**
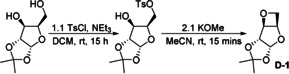
Synthesis of oxetane **D‐1**.

We first investigated the bulk polymerization of **D‐1** using alkali metal alkoxides. KO^*t*^Bu was found to initiate the polymerization of **D‐1** at 150 °C, giving 97 % conversion after 3 h for [**D‐1**]_0_:[KO^*t*^Bu]_0_ loadings of 20:1 (Table 1, entry 1). Lower reaction temperatures resulted in poor conversion of **D‐1** (Table 1, entry 2). Polymers were fully soluble in THF and CHCl_3_ and number average molar masses (*M*_n_), as measured by size exclusion chromatography (SEC), were in good agreement with theoretical values. At 150 °C, *M*_n,SEC_ values of up to 8000 g mol^−1^ could be obtained, although monomer conversion was limited by solidification of the reaction mixture (Table 1, entries 3–4). Initial rate studies indicated first‐order kinetics with respect to monomer concentration, and *M*_n_ was found to increase linearly with conversion (Figures S17–19). At higher conversions however, viscosity may be responsible for mass transfer limitations and deviation from controlled behaviour.

**Table 1:** Polymerization of **D/L‐1**. 
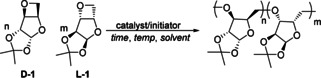

Entry	Monomer **1**	Initiator	[**1**]_0_:[I]_0_	*T* [°C]	*t* [h]	Conv [%]^[a]^	*M*_n,theo_	*M*_n,SEC_ (*Đ*_M_)^[b]^
1	**D‐1**	KO^*t*^Bu	20:1	150	3	97	3600^[c]^	4300 (1.30)
2	**D‐1**	KO^*t*^Bu	20:1	120	3	28	1200^[c]^	–
3	**D‐1**	KO^*t*^Bu	50:1	150	17	69	6200^[c]^	6600 (1.29)
4	**D‐1**	KO^*t*^Bu	100:1	150	17	63	11 100^[c]^	8300 (1.30)
5^[d,e]^	**D‐1**	KO^*t*^Bu/18‐crown‐6	100:1	120	17	77	13 400^[c]^	9200 (1.15)
6^[d]^	**D‐1**	KO^*t*^Bu/18‐crown‐6	200:1	120	22	47	16 300^[c]^	14 500 (1.18)
7^[d]^	**D‐1**	KO^*t*^Bu/18‐crown‐6	400:1	120	22	5	3500^[c]^	8000 (1.15)
8	**D‐1**	KOEt	20:1	150	3	60	2100^[f]^	4000 (1.20)
9	**D‐1**	NaO^*t*^Bu	50:1	150	17	35	3100^[c]^	5000 (1.24)
10^[g]^	**D‐1**	NaO^*t*^Bu/15‐crown‐5	100:1	120	17	28	4900^[c]^	7300 (1.24)
11^[d,h]^	**L‐1**	KO^*t*^Bu/18‐crown‐6	100:1	120	22	51	8900^[c]^	9800 (1.19)
12^[d]^	**D‐1**:**L‐1** (9:1)	KO^*t*^Bu/18‐crown‐6	100:1	120	22	43	7500^[c]^	7900 (1.20)
13^[d]^	**D‐1**:**L‐1** (7:3)	KO^*t*^Bu/18‐crown‐6	100:1	120	22	54	9400^[c]^	10 100 (1.18)
14^[d,i]^	**D‐1**:**L‐1** (1:1)	KO^*t*^Bu/18‐crown‐6	100:1	120	22	61	10 600^[c]^	9700 (1.18)

Reactions carried out in neat **1**. [$\alpha_{D-1}^{25}$ ]=+13.8° [$\alpha_{L-1}^{25}$ ]=−13.2°. [a] Calculated by ^1^H NMR spectroscopy by relative integration of the anomeric protons in **1** (*δ*=6.27 ppm (d, *J=*3.7 Hz)) and poly(**1**) (*δ*=5.88 ppm (d, *J=*3.5 Hz)). [b] Calculated by SEC relative to polystyrene standards using a THF eluent; *Ð*_M_=*M_w_*/*M_n_*. [c] Calculated as *M*_r_(^*t*^BuOH) + (*M*_r_ (**1**) × [**1**]_0_/[I]_0_ × conv/ 100 %). [d] [KO^*t*^Bu]_0_:[18‐crown‐6]_0_=1:1. [e] [$\alpha_{poly(D-1)}^{25}$ ]=−85.3°. [f] Calculated as *M*_r_(EtOH) + (*M*_r_ (**1**) × [**1**]_0_/[I]_0_ × conv/ 100 %). [g] [NaO^*t*^Bu]_0_:[15‐crown‐5]_0_=1:1. [h] [$\alpha_{poly\;(L-1)}^{25}$ ]=+85.8°. [i] [$\alpha_{50:50\;poly(D-1/\;L-1)}^{25}$ ]=−0.6°.

To increase the activity of KO^*t*^Bu, the polymerization was carried out in the presence of 18‐crown‐6. The polymerization then proceeded readily at 120 °C with narrower dispersities (*Đ*_M_, Table 1, entries 5–7). Poly(**D‐1**) with *M*_n,SEC_ of 14 500 g mol^−1^ (*Đ*_M_ 1.15) could be obtained at [**D‐1**]_0_:[KO^*t*^Bu]_0_:[18‐crown‐6]_0_ ratios of 200:1:1 (Table 1, entry 6). Decreasing further the amount of initiator failed to yield higher molar mass polymers, likely due to the presence of protic impurities in the monomer despite distillation (Table 1 entry 7).

**D‐1** was found to be amenable to polymerization with other metal alkoxides at 150 °C, namely KOEt and NaO^*t*^Bu (Table 1, entries 8 and 9). For the former, analysis of the polymer by MALDI‐ToF spectrometry (Table S2) and ^1^H DOSY NMR (Figure S30) spectroscopy confirmed the presence of the ethoxy end groups. The use of 15‐crown‐5 further enhanced the activity of NaO^*t*^Bu, enabling reactions at 120 °C (Table 1, entry 10).

^1^H and ^13^C{^1^H} NMR spectroscopy suggest that the polymerization of **D‐1** using group 1 alkoxides is ring‐selective and highly regioregular (Figure 2 and Figure S6). Comparison of the ^1^H NMR spectra of **D‐1** and poly(**D‐1**) indicates significant conformational changes across the oxetane moiety (Δδ_**c**_=1.35 ppm, Δδ_**d**_=0.83 ppm and Δδ_**e**_=0.85 and 0.65 ppm) and implies selective opening of this ring. The ^13^C{^1^H} NMR spectra of poly(**D‐1**) also features eight well‐defined resonances, adding evidence of head‐to‐tail monomer enchainment. Polarimetry further supports a highly regioselective ROP: poly(**D‐1**) (Table 1, entry 5) exhibits a higher optical rotation ([$\alpha_{poly(D-1)}^{25}$ ]=−85.3°; of opposite sign to **D‐1**) than reported previously ([$\alpha_{poly(D-1)}^{25}$ ] up to −81.3°).^[13]^ Compared with cationic methods,^[13]^ in addition to improved conversions, excellent *M*_n_ control and more accessible reaction conditions, anionic polymerization hence also appears to limit side reactions and improve regioregularity.

**Figure 2:**
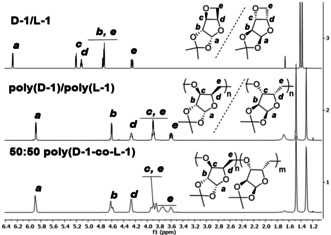
^1^H NMR (CDCl_3_, 25 °C) spectra of **D‐1** and **L‐1** (top), of poly (**D‐1**) and poly (**L‐1**) (middle), and of poly (**D‐1**‐*co*‐**L‐1**).

DFT modelling confirms these conclusions (Figure 3 and Figure S48). A strong kinetic preference for KO^*t*^Bu initiation to occur at the oxetane and expose the secondary hydroxy (***e*** **‐O′**) was calculated (ΔΔ*G* ^≠^_TS1_ ***e*** **‐O′**=+26.3 kcal mol^−1^; vs. +43.4, +43.4, and +48.6 kcal mol^−1^ for ***c*** **‐O′**, ***a*** **‐O′′** and ***a*** **‐O′′′** openings, respectively). The ***e*** **‐O′** opening is also thermodynamically favoured compared to other possibilities (ΔΔ*G**e*** **‐O′**=−16.3 kcal mol^−1^; vs. −13.7, +0.3, and +0.8 kcal mol^−1^ for ***c*** **‐O′**, ***a*** **‐O′′** and ***a*** **‐O′′′** products, respectively). Propagation was also modelled and the kinetic and thermodynamic selectivity for opening at the ***e*** position is maintained (Figure S48), suggesting Head‐to‐Tail enchainment, as insinuated by NMR spectroscopy.

**Figure 3:**
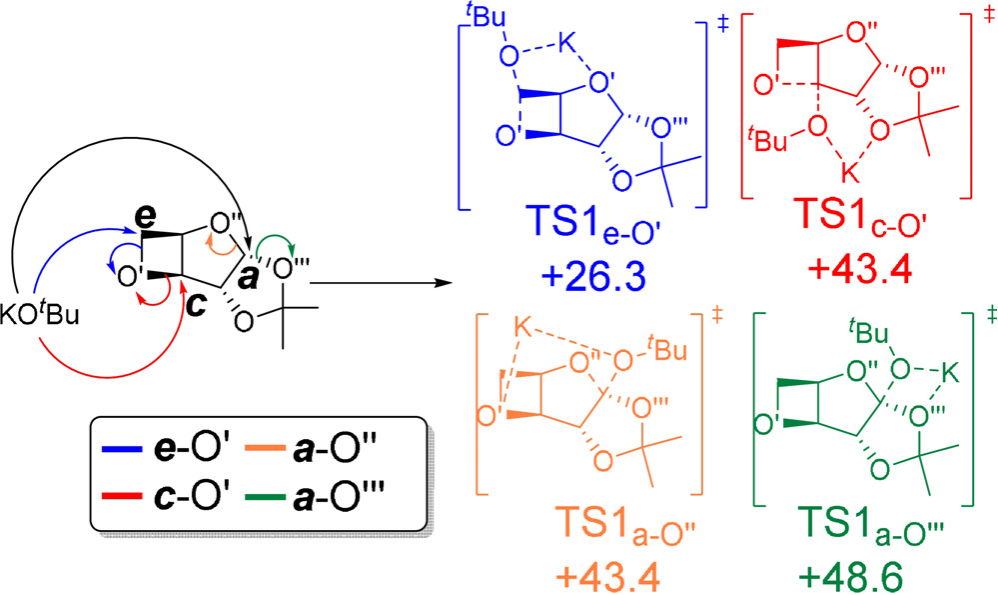
DFT‐computed barriers (ΔΔ*G* ^≠^_,_ in kcal mol^−1^) for the opening of **D‐1** with KOtBu with ωb97xD functional, basis sets 6–311++G(2d,p) (O, K) and 6–31+G(d,p) (C, H), solvent model: cpcm=THF and temperature=423 K.

Similarly, **L‐1** was synthesized from l‐xylose and polymerized, with poly(**L‐1**) displaying identical NMR spectra to poly(**D‐1**) but opposite specific rotation (Table 1, entry 11; Table S4). One‐pot copolymerizations were also carried out with various [**D‐1**]_0_:[**L‐1**]_0_ ratios (Table 1, entries 12–14). Compared with the homochiral polymers, the heterochiral copolymers show broader and new resonances between 3.75 and 3.80 ppm in the ^1^H NMR spectrum (Figure 2 and Figure S9). We believe that these new signals correspond to new *racemo* diads, consistent with the statistical incorporation of both enantiomers in the polymers. Polarimetry measurements show that the 50:50 poly(**D‐1**‐*co*‐**L‐1**) is achiral, supporting further a lack of tacticity.

Thermogravimetric analysis (TGA) of poly(**D‐1**), poly(**L‐1**) and 50:50 poly(**D‐1**‐*co*‐**L‐1**) of approximately 10 000 g mol^−1^ indicated a degradation onset temperature (*T*_d,onset_) of above 300 °C for all, with a single degradation step (Figures S31–33). 50:50 Poly(**D‐1**‐*co*‐**L‐1**) was found to be less thermally stable than both poly(**D‐1**) and poly(**L‐1**) with lower *T*_d,onset_ (301 °C, 315 °C and 318 °C, respectively) and temperature of 5 % mass loss (*T*_d5_, 342 °C, 348 °C and 355 °C).

Differential scanning calorimetry (DSC) showed that the homochiral polymers are semi‐crystalline, with a *T*_g_ between 131–135 °C and a melting temperature (*T*_m_) between 271–281 °C (Figures S37,38). Wide Angle X‐ray Scattering (WAXS) analysis confirmed the presence of crystalline domains (Figure S45). Homochiral poly(**1**) have remarkable thermal properties, with a *T*_g_ much higher than any other aliphatic polyethers,^[7]^ similar to known sugar‐derived polycarbonates,[^2a^, ^2b^, ^11b^, ^15^] but are crystalline and significantly more thermally robust. Heterochiral 50:50 poly(**D‐1**‐*co*‐**L‐1**) was found to be amorphous with a clear glass transition at 128 °C (Figure S42). In fact, incorporation of even 10 % of **L‐1** disrupted the crystallinity of poly(**D‐1**) (Figures S40,41).

We also attempted to further enhance the properties of the homochiral polymers by exploiting stereocomplexation. Solutions of poly(**D‐1**) and poly(**L‐1**) in CHCl_3_ were mixed, the solvent slowly evaporated and the samples annealed under vacuum for 24 hours at 100 °C. TGA revealed no significant differences between the homopolymers and 50:50 and 75:25 polymer blends (Figures S34,S35). However, DSC showed an increase in melting temperature for the 50:50 blend (288 °C, Δ*T*_m_ of +6–17 °C compared with the parent polymers, Figure 4 and Figure S43). In contrast, three melting transitions were detected for the 75:25 blend, (Figure S44), indicative of multiple crystalline phases. Conversely to the homochiral polymers, WAXS analysis of the stereocomplex displayed broad signals (Figure S47), indicative of significant amorphous regions in the material or of very small crystalline domains. The co‐crystallization of poly(**D‐1**) and poly(**L‐1**) remains to be optimized. However, to the best of our knowledge, this is the first time that a stereocomplex of a polysaccharide mimic is reported.

**Figure 4:**
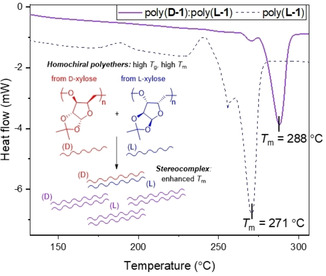
DSC thermograms showing the 2^nd^ heating cycle (20 °C min^−1^) for poly(**L‐1**) (dashed, blue; from Table entry 11) and 50:50 blend of poly(**D‐1**) and poly(**L‐1**) (purple; from Table 1 entries 5 and 11, respectively).

Controlled deprotection of the acetal groups in poly(**D‐1**) and poly(**L‐1**) could be performed by acid hydrolysis, revealing up to 97 % of the xylose unit hydroxyl groups (Figures S20–S22). This led to a significant decrease in the thermal stability of the polymer (*T*_d5_=168 °C, Figure S36), and no glass transition or crystallinity detectable. Above 28 % of deprotection, SEC analysis revealed the presence of several high *M*_n_ species, indicative of aggregation in solution, which however disappeared upon gentle heating of the samples (exposing polymers of expected *M*_n_; consistent with the hydrolytic stability of the ether linkages), then reformed over several days (Figure S28). The rapid dissociation of the aggregates upon heating suggests reversible H‐bonding between chains. At deprotection levels of 44 % and above, the polymers were found to be water soluble. We also showed that the hydroxyl groups were amenable to further functionalization, for example, by reaction with chlorodiphenylphosphine, while keeping the polymer chain intact (Figures S49–S52).

In conclusion, the chirality of xylose has been exploited to modulate the properties of a family of carbohydrate polymers. Produced by controlled anionic ROP of d‐ or l‐anhydrosugar derivatives, the homochiral and isotactic polyethers are semicrystalline, whilst statistical copolymerization of both enantiomers yields an atactic, amorphous material. This renewable monomer system may offer a new platform for the development of stereoselective catalysts, and the renewable materials with high *T*_g_ and *T*_m_ may find applications as hard blocks of thermoplastic elastomers. We also report the formation of a novel stereocomplex, with enhanced thermal properties compared with its homochiral parents. Revealing the hydroxyl groups of these polysaccharide mimics enables reversible cross‐linking, with potential applications in self‐healing materials, as well as post‐polymerization functionalization, for example, for the synthesis of chiral macromolecular catalysts. Future studies will also focus on the impact of polymer tacticity and the presence of an unnatural sugar on the biocompatibility and biodegradation of these materials.

## Conflict of interest

The authors declare no conflict of interest.

## Supporting information

As a service to our authors and readers, this journal provides supporting information supplied by the authors. Such materials are peer reviewed and may be re‐organized for online delivery, but are not copy‐edited or typeset. Technical support issues arising from supporting information (other than missing files) should be addressed to the authors.

Supplementary
