# Supplementary material for: Control of Crystallinity and Stereocomplexation of Synthetic Carbohydrate Polymers from d‐ and l‐Xylose
Source: Angew Chem Int Ed Engl. 2021 Jan 7;60(9):4524–8. doi: 10.1002/anie.202013562 (PMC7986207; doi:10.1002/anie.202013562)
Supplement: Supplementary file 1 — Supplementary [file ANIE-60-4524-s001.pdf]

Angewandte Chemie
Eine Zeitschrift der Gesellschaft Deutscher Chemiker

Supporting Information

Control of Crystallinity and Stereocomplexation of Synthetic Carbohydrate Polymers from D- and L-Xylose

*Thomas M. McGuire, Jessica Bowles, Edward Deane, Elliot H. E. Farrar, Matthew N. Grayson, and Antoine Buchard**

anie_202013562_sm_miscellaneous_information.pdf

Table of Contents

Table of Contents 2
Experimental Procedures 3
1. Materials and Methods 3
2. General Procedures 4
2.1. Synthesis of 1,2-O-Isopropylidene-xylofuranose (IPXF) 4
2.2. Synthesis of 1,2- O-isopropylidene-5-O-tosyl-xylofuranose (Ts-IPXF) 4
2.3. Synthesis of **D-1** or **L-1** 4
2.4. General procedure for the homopolymerization of **D-1** or **L-1** 4
2.5. General procedure for the stereocomplexation of poly(**L-1**) and poly(**D-1**) 5
2.6. General procedure for the deprotection of poly(**D-1**) 5
2.7. General procedure for the phosphorylation of poly(**D-1**) 5
3. Characterization of compounds 6
4. Kinetics 14
4.1. Polymerization kinetics 14
4.2. Deprotection kinetics 16
5. Additional polymerization results 18
6. $^{1}H\{^{1}H\}$ NMR spectroscopy 19
7. SEC chromatograms 20
8. End group analysis 23
9. Thermal characterization 25
9.1. TGA traces 25
9.1.1. Homochiral and heterochiral polymers 25
9.1.2. Blends 26
9.1.3. Deprotected polymer 27
9.2. DSC traces 28
9.2.1. Homochiral polymers 28
9.2.2. Heterochiral polymers 29
9.2.3. Blends 31
10. Wide-angle X-ray scattering (WAXS) analysis 33
11. DFT 35
12. Phosphorylation of deprotected poly(**D-1**) 38
13. Optical rotation data 40
References 41
Author Contributions 41

Experimental Procedures

1. Materials and Methods

All manipulations were performed under an atmosphere of argon using standard Schlenk techniques unless otherwise stated. All reagents were purchased from either Merck, Alfa Aesar, Acros Organics or Carbosynth and used without further purification. All solvents used were anhydrous unless otherwise stated.

NMR spectra were recorded on a Bruker 400 or 500 MHz instrument and referenced to residual solvent peaks. Coupling constants are given in Hertz. Polymerization conversions were determined by ^{1}H NMR spectroscopy (see details in caption of Table 1, main text).

Size-exclusion chromatography (SEC) was carried out using either a THF or DMF eluent. Multi analysis software was used to process the data. Polymer samples were dissolved at a concentration of 2 mg mL^{-1}.
THF samples were recorded on an Agilent 1260 Infinity series instrument at 1 mL min^{-1} at 35 °C using two PLgel 5 μm MIXED-D 300 × 7.5 mm columns in series. Samples were detected with a differential refractive index (RI) detector. Number-average molecular weight ($M_{n,SEC}$), and dispersities, ($Đ_M$ (M_w/M_n)) were calculated against a polystyrene calibration (11 polystyrene standards of narrow molecular weight, ranging from M_w 615 – 568000 Da).
DMF samples were measured on an Agilent 1260 SEC MDS instrument at 0.5 mL min^{-1} at 50 °C using a Polargel-M 300 x 7.5 mm column. Samples were detected using a RI detector. $M_{n,SEC,}$ and $Đ_M$ were calculated against a polymethylmethacrylate calibration (11 polymethylmethacrylate standards of narrow molecular weight ranging from M_w 885 – 260900).

Differential scanning calorimetry (DSC) was carried out using a MicroSC multicell calorimeter from Setaram; the Calisto program was employed to collect and process the data. Thermograms were plotted using Origin 2018 graphing software. The measurement cell and the reference cell were both a 1 mL Hastelloy C cell; a mass of 2–5 mg of polymeric material was loaded into the measurement cell with the reference cell empty. The experiments were performed under N_2 and the sample heated and cooled at a rate of 20 K min^{-1} unless otherwise stated. A second heating and cooling cycle was carried out immediately following completion of the first, unless otherwise stated.

Thermogravimetric analysis (TGA) was carried out using A Setsys Evolution TGA 16/18 from Setaram; the Calisto program was employed to collect and process the data. TGA traces were plotted using Origin 2018 graphing software. The sample was loaded into a 170 µL alumina crucible and the analytical chamber purged with argon (200 mL min^{-1}) for 40 min prior to starting the analysis. The sample was then heated under an argon flow (20 mL min^{-1}) from 30 to 600 °C at a rate of 10 °C min^{-1}, unless otherwise stated

Mass spectrometry measurements were recorded with a microToF electrospray time-of-flight (ESI-ToF) mass spectrometer (Bruker Daltonik) in acetonitrile.

Matrix-assisted laser desorption ionization-time of flight (MALDI-ToF) mass spectrometry was conducted using a Bruker Autoflex speed MALDI Mass Spectrometer equipped with a 2 kHz Smartbeam-II laser. 2.4 mg of dithranol (DT) was dissolved in 0.125 mL of chloroform in glass vial to make a solution of the matrix. 1.0 mg of polymer analyte was dissolved in 0.45 mL of chloroform in a glass vial to make a solution of sample. 20µL of matrix solution and 10µL of sample solution were pipette mixed together in an Eppendorf and 0.75µL of the mixture was spotted onto the target section of a 384 well ground steel MALDI-TOF plate. The spot was left to dry for approximately 5 min. before being inserted into the MALDI instrument for analysis. *Note that no cationising agent (salt) is added to the MALDI mixture.*
Once loaded, positive ion MALDI spectra were obtained in linear and reflector mode. Laser intensity was varied. The data was analysed using the Flex Analysis software, version 3.4 (build 76). The molecular weight distributions were obtained through analysis of the data in the Polytools software package 1.31.

Wide angle X-Ray scattering (WAXS) experiments were conducted using a SAXSpoint 2.0 by Anton Paar, equipped with a microfocus X-ray source (Cu, 50 W) and a EIGER R 1M detector. Samples were loaded on to a multi-solid sampler mounted on a vario-stage and held in place by tape. Data was processed using SAXSAnalysis by Anton Paar. Blank scattering profiles were subtracted prior to the data being plotted with Origin 2018.

Optical rotation measurements were recorded at 25 °C in $CHCl_3$ solutions (concentrations 2–8 mg mL^{-1}) using an MCP 150 modular compact polarimeter by Anton PaarMain Text Paragraph.

2. General Procedures

2.1. *Synthesis of 1,2-O-Isopropylidene-xylofuranose (IPXF)*

Following an adapted literature procedure:[1] to a round bottom flask was charged D- or L-xylose (20.00 g, 133.34 mmol, 1.00 equiv.), non-anhydrous acetone (500 mL) and 95–98% concentrated H_2SO_4 (20 mL, 373.16 mmol, 2.80 equiv.) forming a pale-yellow suspension. The suspension was left to stir until the solid had fully dissolved to form a yellow solution (*ca* 1 h). Upon full dissolution, the reaction mixture was cooled over ice and an aqueous solution of K_2CO_3 (224 mL, 1.10 mol L^{-1}, 246.01 mmol, 1.84 equiv.) was slowly added. The resultant white suspension was allowed to stir at room temperature with aliquots taken at regular intervals to monitor the deprotection by TLC (1:1 $HCCl_3$:acetone eluent) and NMR (d^6-DMSO). After 2.5 h near quantitative formation of the monoprotected sugar was observed. Solid K_2CO_3 (17.80 g, 128.79 mmol, 1.04 equiv.) was added slowly at room temperature and the pH adjusted until 7–8 by litmus paper. The suspension was then filtered, and the acetone removed *in vacuo* at 40 °C. The aqueous phase was washed with DCM (100 mL, x 3) and the organic phases were collected and back extracted with water (50 mL, x 3). The aqueous phases were collected, and the water removed *in vacuo* at 50 °C. The resultant oil was dissolved in EtOAc and stirred over $MgSO_4$ overnight. The suspension was filtered, and the solvent removed *in vacuo* at 40 °C to yield 1,2-*O*-Isopropylidene-α-xylofuranose (**IPXF**) as a clear oil (22.81 g, 90% yield).

2.2. *Synthesis of 1,2- O-isopropylidene-5-O-tosyl-xylofuranose (Ts-IPXF)*

Following an adapted literature procedure:[2] **IPXF** (10.00 g, 52.58 mmol, 1.00 equiv.) and tosyl chloride (11.03 g, 57.83 mmol, 1.10 equiv.) were charged to a round-bottomed flask and dissolved in dichloromethane (100 mL). The reaction vessel was cooled over ice and triethylamine (50 mL, 286.98 mmol, 5.46 equiv.) was added. The vessel was left to warm to 20 °C over 15 hours. Ethyl acetate (150 mL) was then charged to the vessel and the mixture was transferred to a separating funnel. The organic phase was then washed with brine (50 mL, × 1), 1 M sodium hydrogen carbonate (50 mL, × 1) and water (50 mL, × 1). The organic phase was collected and dried over magnesium sulfate, filtered and concentrated *in vacuo* at 40 °C to give a white solid. The solid was then stirred over cold Et_2O (–18 °C, 50 mL) for 10 minutes, filtered over a glass frit and rinsed again with cold Et_2O (50 mL). The precipitate was left to dry in a vacuum oven overnight at 50 °C to give the product as a white solid (13.90 g, 40.49 mmol, 77% yield).

2.3. *Synthesis of D-1 or L-1*

To a Schlenk flask was charged **Ts-IPXF** (10.97 g, 31.85 mmol, 1.00 equiv.) and anhydrous acetonitrile (100 mL). The suspension was agitated at 400 rpm until a clear colourless homogenous solution was obtained. KOMe (4.69 g, 66.89 mmol, 2.10 equiv.) was charged to the solution and stirred for 15 minutes, after which the reaction was quenched by addition of water (30 mL). The acetonitrile was removed *in-vacuo* at 40 °C. The aqueous phase was extracted with Et_2O (50 mL, × 3). The organic phases were collected and washed with brine (50 mL, × 1), 1 M sodium hydrogen carbonate (50 mL, × 1) and water (50 mL, × 1). The organic phases were collected, dried over $MgSO_4$, filtered and concentrated *in vacuo* at 40 °C to give **D-1** (5.00 g, 29.04 mmol, 91% yield) as a clear oil. For further purification, the oil was stirred over CaH_2 at 80 °C overnight and vacuum distilled (1×10^{-2} mbar, 40 °C) to yield the oxetane as a clear oil (4.00 g, 23.25 mmol, 1.00 equiv., 73% yield).

2.4. *General procedure for the homopolymerization of D-1 or L-1*

To a 2 mL dram vial under argon was charged **D-1** (344 mg, 200.00 equiv.), KO*t*Bu (20 µL, 0.5 mol L^{-1} in THF, 1.00 equiv.) and 18-crown-6 (20 µL, 0.5 mol L^{-1} in THF, 1.00 equiv.). The vial was sealed and heated to 120 °C with stirring. After 22 hours the vial was cooled and the polymerization was quenched with a DCM solution of benzoic acid (0.1 mL, 10 mg mL^{-1}). The solid was dissolved in the minimum amount of $CHCl_3$ then precipitated from cold Et_2O. The suspension was centrifuged (2900 rpm, 5 minutes) and the solid phase was collected. The polymer was then re-dissolved in $CHCl_3$ and reprecipitated twice more from cold Et_2O with centrifugation (2900 rpm, 5 minutes) The solid was collected and dried in a vacuum oven for 24 hours at 100 °C to yield the polyether (239 mg, 69% yield).

*For co-polymerizations of **D-1** and **L-1**, the procedure is identical to above except that the polymer is precipitated from hexane.*

2.5. General procedure for the stereocomplexation of poly(L-1) and poly(D-1)

To a dram vial was added poly(**D-1**) (25 mg) and poly(**L-1**) (25 mg). The polymer samples were dissolved in $HCCl_3$ and allowed to stir for 30 minutes. The solution was then evaporated over a stream of air. The solid phase was dried in a vacuum oven at 100 °C for 24 hours to yield the stereocomplex (50 mg, 100% yield).

2.6. General procedure for the deprotection of poly(D-1)

Following an adapted literature procedure:[3] to a dram vial was added poly(**D-1**) (100 mg) and DCM (0.4 mL). The solution was agitated until fully dissolved. Upon dissolution, the reaction mixture was cooled over ice and a 4:1 TFA:H_2O solution (1.0 mL) was added. At predetermined intervals, aliquots were taken from the reaction solution and precipitated from cold Et_2O to determine the amount of deprotection by ^{1}H NMR spectroscopy. After 8 h, cold Et_2O was added to the reaction mixture and the suspension was centrifuged (2900 rpm, 5 minutes). The solid phase was collected and rinsed twice more in cold Et_2O or until the supernatant was neutral by litmus test. The solid phase was dried in a vacuum oven at 100 °C for 24 hours to yield poly(**D-1**) deprotected at 85% (71 mg, 90% yield*)

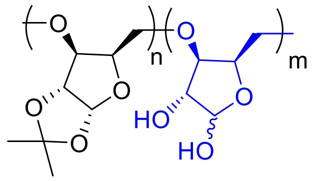

2.7. General procedure for the phosphorylation of poly(D-1)

Following an adapted literature procedure:[4] to a dram vial under argon was charged poly(**D-1**) deprotected at 28% (40 mg, *ca* 0.25 mmol**), 4-dimethylamine pyridine (61 mg, 0.50 mmol, 2.00 equiv.) non-anhydrous NEt_3 (0.310 mL, 2.25 mmol, 9.00 equiv.) and THF (2 mL). The suspension was agitated until fully dissolved. PPh_2Cl (0.115 mL, 0.63 mmol, 2.50 equiv.) was then added under a stream of argon resulting in immediate precipitation of a white solid. The suspension was left to stir for a further 68 h after which the solvent was removed *in vacuo* at 40 °C. The resultant solid was dissolved in the minimum amount of DCM and precipitated from cold Et_2O. The suspension was then centrifuged (2900 rpm, 5 minutes) and the solid phase was collected. The polymer was then re-dissolved in DCM and reprecipitated twice more from cold Et_2O with centrifugation (2900 rpm, 5 minutes). The solid was collected and dried in a vacuum oven for 24 hours at 100 °C to yield the partially (26%) phosphorylated polymer (50 mg, 76% yield*). SEC analysis indicated that the polymer chain had remained intact (Figures S44–S47), while ^{31}P NMR spectroscopy revealed the oxidation of the phosphorus atoms during functionalization (Figure S45), probably due to residual moisture.

*yield calculated in terms of moles of monomeric species, e.g. for deprotection:
Maximum mmol of deprotected polymer = 100/M_r D-1 = 100/172.14 = 0.581 mmol
Maximum mass of polymer = 0.581[0.15(M_r **D-1**)+0.85 (M_r 100% deprotected **D-1**)]
= 0.581[0.15(172.14)+0.85 (130.06)]:
= 79 mg
Yield = 71/79 = 90%.

**mmol calculated as:
mass of material/[0.72(M_r **D-1**)+0.28 (M_r 100% deprotected **D-1**)]:
40 mg/[0.72(172.14)+0.28(130.06)]
= 0.25 mmol

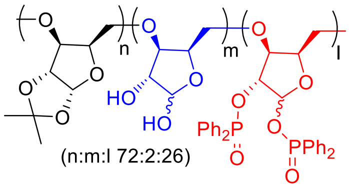

3. Characterization of compounds

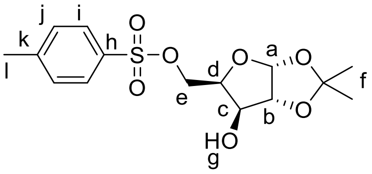

Ts-IPXF:

White solid: 77% yield (13.90 g).

^{1}H NMR (400 MHz, Chloroform-*d*) δ 7.80 (d, *J* = 8.4 Hz, 2H, ***H$_i$***), 7.36 (d, *J* = 8.1 Hz, 2H, ***H$_j$***), 5.87 (d, *J* = 3.6 Hz, 1H, ***H$_a$***), 4.51 (d, *J* = 3.6 Hz, 1H, ***H$_b$***), 4.35 (t, *J* = 6.4 Hz, 1H, ***H$_e$***), 4.33 (d, *J* = 2.4 Hz, 1H, ***H$_d$***), 4.30 (d, *J* = 5.4 Hz, 1H, ***H$_c$***), 4.19 – 4.06 (m, 1H, ***H$_e$***), 2.45 (s, 3H, ***H$_l$***), 2.40 (s, 1H, ***H$_g$***), 1.46 (s, 3H, ***H$_f$***), 1.30 (s, 3H, ***H$_f$***);

^{13}C {^{1}H} NMR (101 MHz, Chloroform-*d*) δ 145.4 (***C$_h$***), 132.6 (***C$_k$***), 130.1 (***C$_i$***), 128.2 (***C$_j$***), 112.3 (***C$_{acetal}$***), 105.1 (***C$_a$***), 85.2 (***C$_b$***), 77.8 (***C$_d$***), 74.5 (***C$_c$***), 66.4 (***C$_e$***), 26.9 (***C$_f$***), 26.4 (***C$_f$***), 21.8 (***C$_l$***), in accordance with literature.[5]

MS (ESI): $[C_{15}H_{20}O_7S_1+Na]^+$ Theo. 367.0822, found 367.0849.

$[\alpha_D^{25}]$ = +13.6 ° $[\alpha_L^{25}]$ = –12.0 °(concentration = 2 mg mL^{-1}).

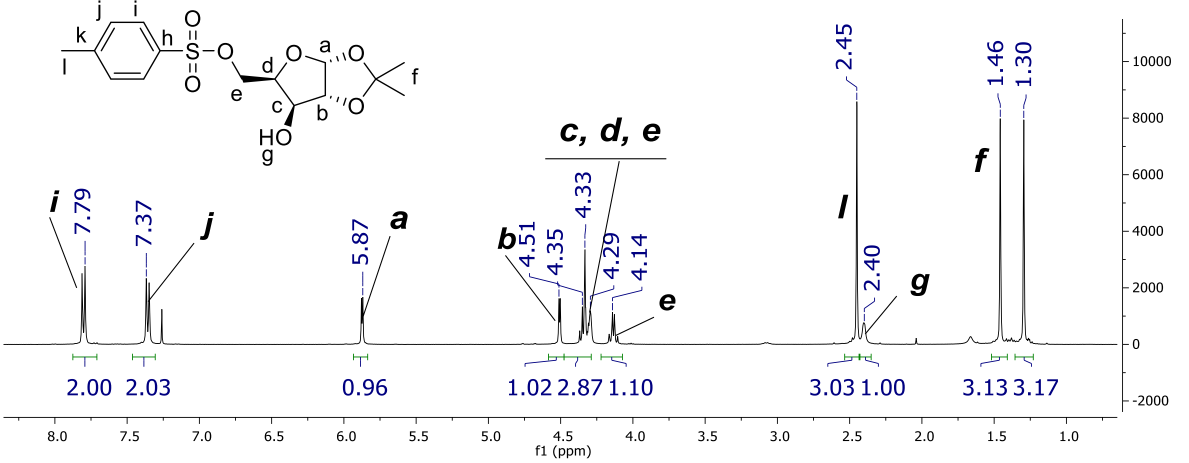

Figure S1. ^{1}H NMR spectrum (400 MHz, $CDCl_3$) of **Ts-IPXF** ($CHCl_3$ residual signal at 7.26 ppm).

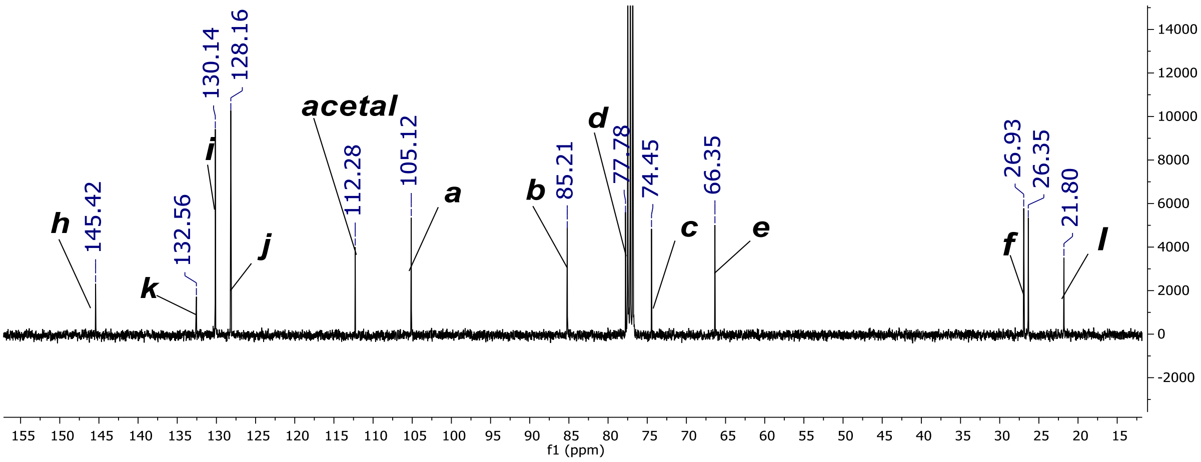

Figure S2. ^{13}C{^{1}H} NMR spectrum (400 MHz, $CDCl_3$) of **Ts-IPXF** ($CHCl_3$ residual signal at 77.2 ppm).

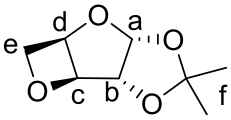

D-1/L-1:

Clear oil; 73% yield (4.00 g).

^{1}H NMR (400 MHz, Chloroform-*d*) δ 6.27 (d, *J* = 3.7 Hz, 1H, ***H**a*), 5.19 (d, *J* = 4.0 Hz, 1H, ***H**c*), 5.10 (td, *J* = 4.1, 2.2 Hz, 1H, ***H**d*), 4.74 (d, *J* = 4.3 Hz, 1H, ***H**e*), 4.72 (d, *J* = 4.0 Hz, 1H, ***H**b*), 4.25 (dd, *J* = 7.7, 2.3 Hz, 1H, ***H**e*), 1.40 (s, 3H, ***H**f*), 1.37 (s, 3H, ***H**f*);

^{13}C{^{1}H} NMR (101 MHz, Chloroform-*d*) δ 114.0 (***C**acetal*), 108.3 (***C**a*), 87.7 (***C**c*), 84.8 (***C**b*), 78.6 (***C**e*), 78.4 (***C**d*), 28.0 (***C**f*), 27.3 (***C**f*), in accordance with literature.[6]

MS (ESI): [$C_8H_{12}O_4$+H]$^+$ Theo. 173.0808, found 173.0808.

[α_D^{25}] = +13.8 ° [α_L^{25}] = –13.2 ° (concentration = 8 mg mL^{-1}).

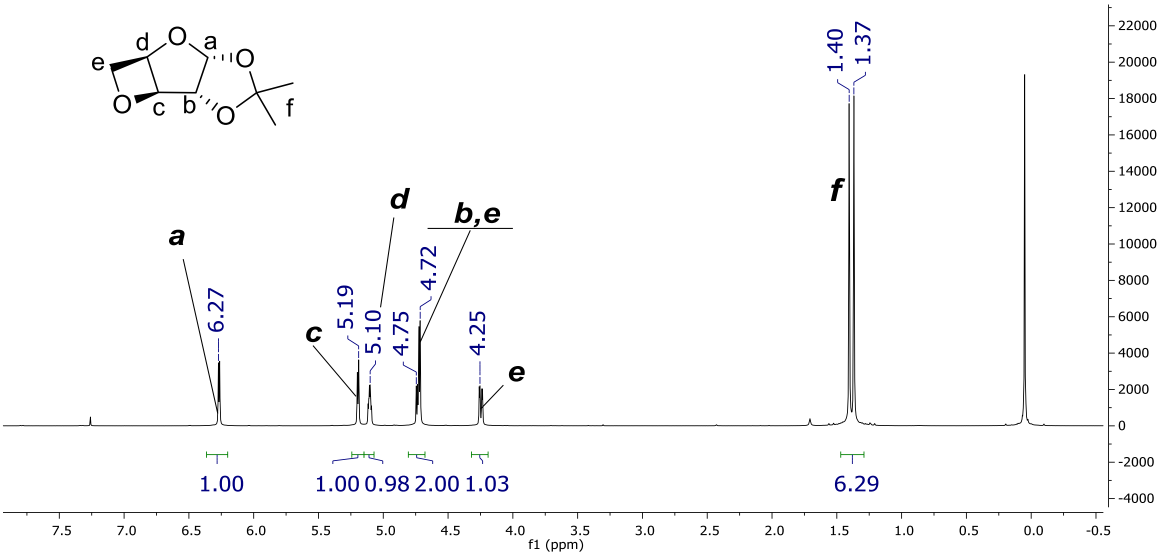

Figure S3. ^{1}H NMR spectrum (400 MHz, $CDCl_3$) of **D-1** ($CHCl_3$ residual signal at 7.26 ppm).

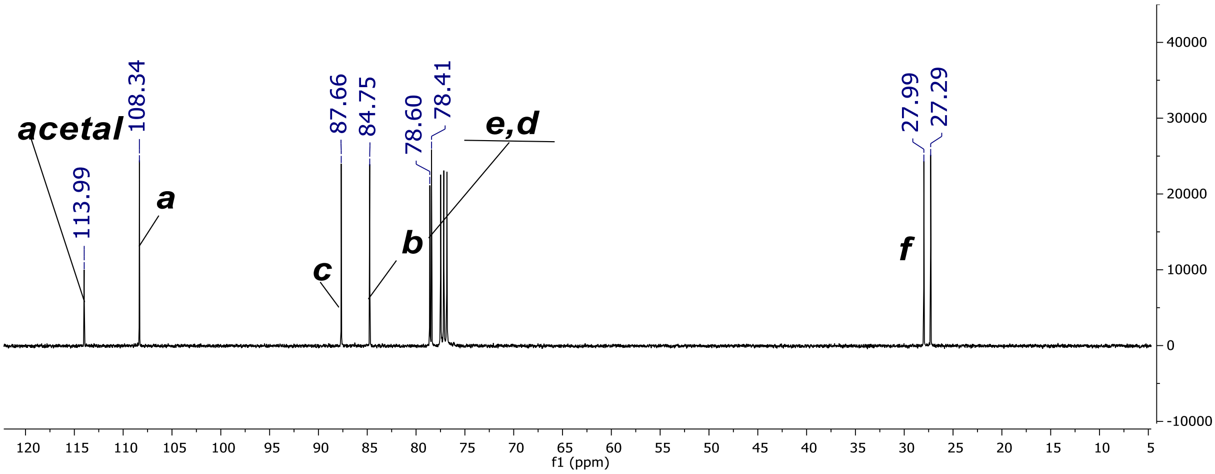

Figure S4. ^{13}C{^{1}H} NMR spectrum (101 MHz, $CDCl_3$) of **D-1/L-1** ($CHCl_3$ residual signal at 77.16 ppm).

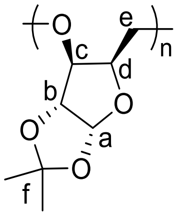

Poly(D-1)/ poly(L-1):

Off-white powder (69% yield, 239 mg).

^{1}H NMR (500 MHz, Chloroform-*d*) δ 5.88 (d, *J* = 3.5 Hz, 1H, ***H$_a$***), 4.60 (d, *J* = 3.5 Hz, 1H, ***H$_b$***), 4.27 (td, *J* = 7.1, 6.1, 3.7 Hz, 1H, ***H$_d$***), 3.89 (m, 2H, ***H$_c$*** and ***H$_e$***), 3.60 (dd, *J* = 10.3, 6.8 Hz, ***H$_e$***), 1.48 (s, 3H, ***H$_f$***), 1.31 (s, 3H, ***H$_f$***);
^{13}C{^{1}H} NMR (126 MHz, Chloroform-*d*) δ 111.7 (***C$_{acetal}$***), 105.3 (***C$_a$***), 83.1 (***C$_c$***), 81.9 (***C$_b$***), 79.4 (***C$_d$***), 68.0 (***C$_e$***), 27.0 (***C$_f$***), 26.4 (***C$_f$***). In accordance with the literature[7]

$M_{n,SEC}$ = 9200 g mol^{-1}, $Đ_M$ = 1.30; T_g 134 °C; T_m = 271 – 281 °C; T_c = 193 – 248 °C; $T_{d,onset}$ = 315 – 318 °C.

$[\alpha^{25}_{poly(D-1)}]$ = –85.3°; $[\alpha^{25}_{poly\ (L-1)}]$ = +85.8 °; $[\alpha^{25}_{poly(D-1)+poly(L-1)}]$ = +0.5° (concentration = 2 mg mL^{-1}).

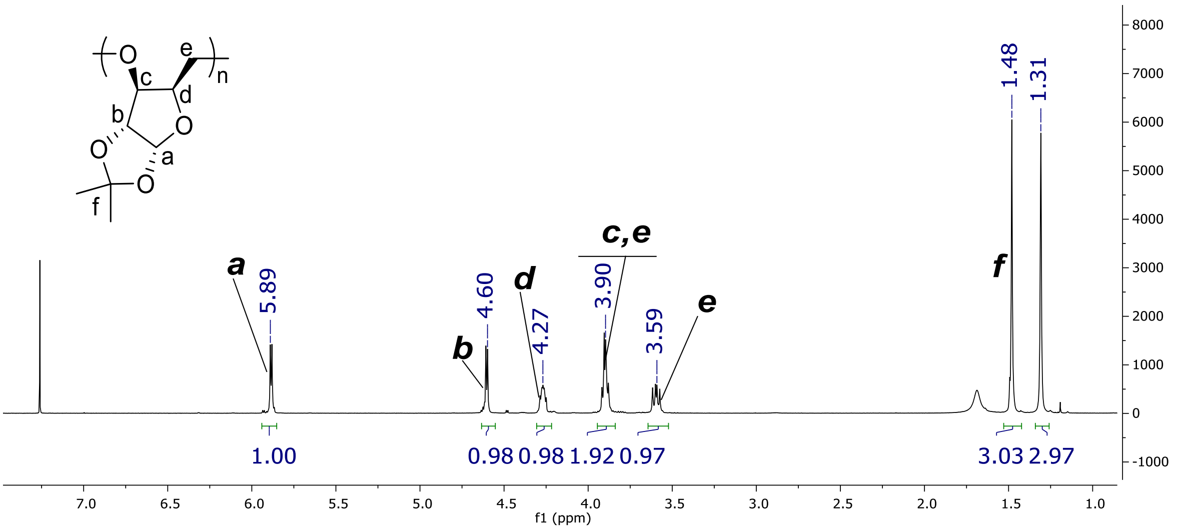

Figure S5. ^{1}H NMR spectrum (500 MHz, $CDCl_3$) of poly(**D-1**)/poly(**L-1**) $CHCl_3$ residual signal at 7.26 ppm).

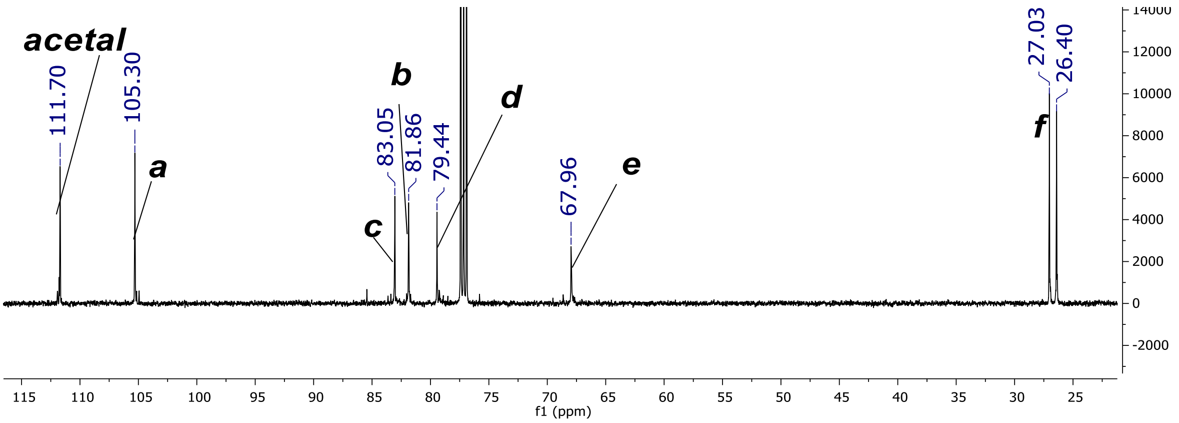

Figure S6. ^{13}C{^{1}H} NMR spectrum (126 MHz, $CDCl_3$) of poly(**D-1**)/poly(**L-1**) ($CHCl_3$ residual signal at 77.2 ppm).

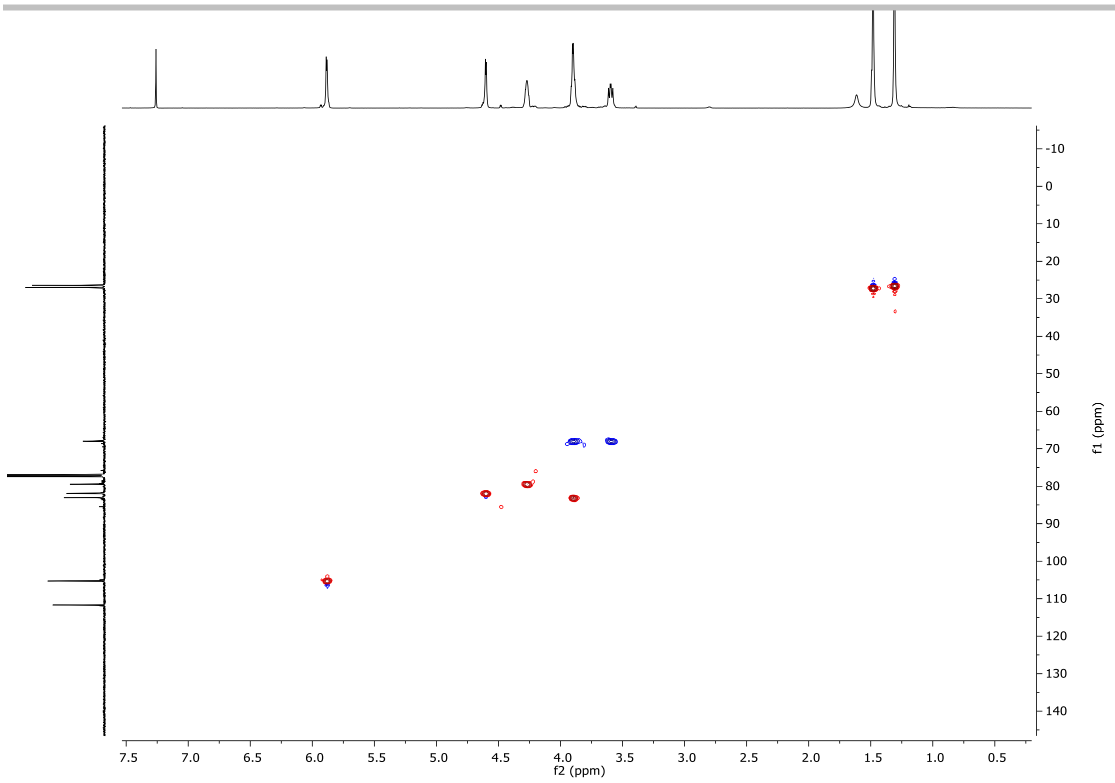

Figure S7. $^{13}C\{^{1}H\}$ HSQC NMR spectrum ($CDCl_3$) of poly(D-1).

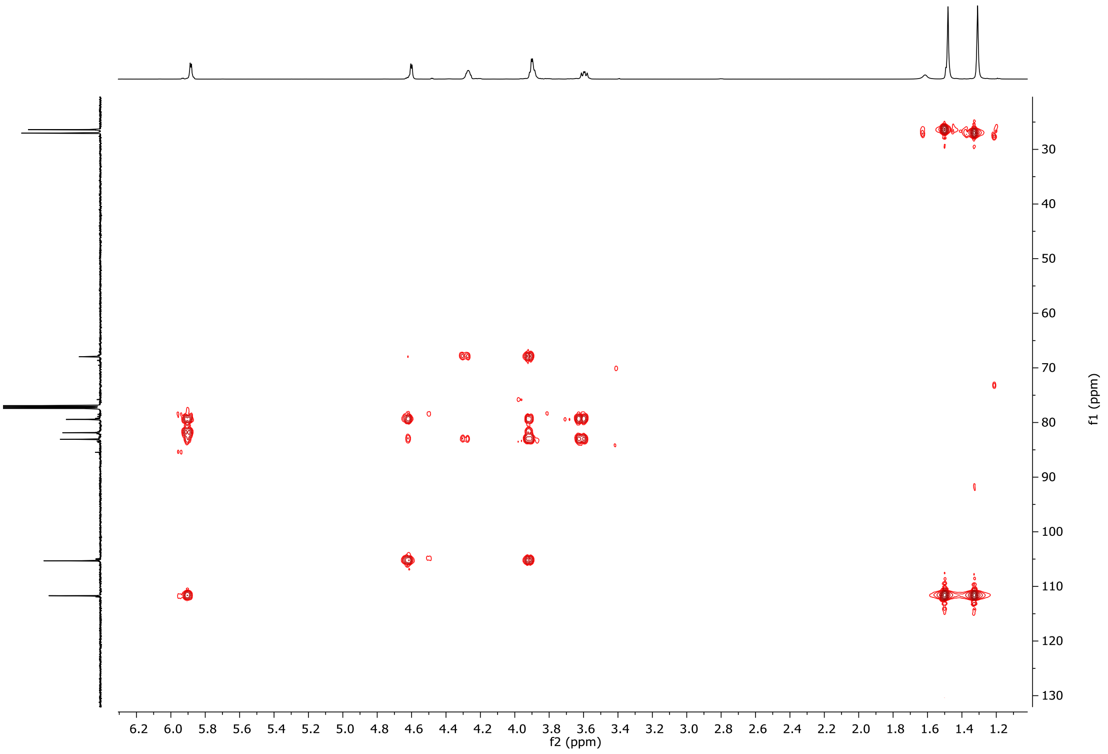

Figure S8. 1H- 13C{1H} HMBC NMR spectrum (CDCl3) of poly(D-1).

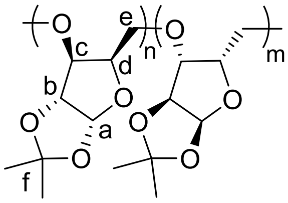

50:50 Poly (D-1-*co*-L-1):

Off-white powder, 74% yield (255 mg).

^{1}H NMR (500 MHz, Chloroform-*d*) δ 5.95 – 5.81 (m, 1H, H_a), 4.71 – 4.51 (m, 1H, H_b), 4.35 – 4.19 (m, 1H, H_d), 3.99 – 3.82 (m, 2H, H_c and H_e), 3.80 – 3.69 (m, 1H, H_e), 3.66 – 3.51 (m, 1H, H_e), 1.48 (s, 3H, H_f), 1.30 (s, 3H, H_f);
^{13}C{^{1}H} NMR (126 MHz, Chloroform-*d*) δ 111.7 (C_{acetal}) , 105.3 (C_a), 84.7 – 82.5 (C_c), 82.8 – 81.3 (C_b), 80.2 – 78.3 (C_d), 69.4 – 66.7 (C_e), 27.0 (C_f), 26.4 (C_f).

M_{nSEC} = 15000; $Đ_M$ = 1.11; T_g 134 °C; T_m = *not observed*; T_c = *not observed*; $T_{d,onset}$ = 301 °C.

$[\alpha^{25}_{50:50\ poly(D-1/\ L-1)}]$ = –0.6 ° (concentration = 2 mg mL^{-1}).

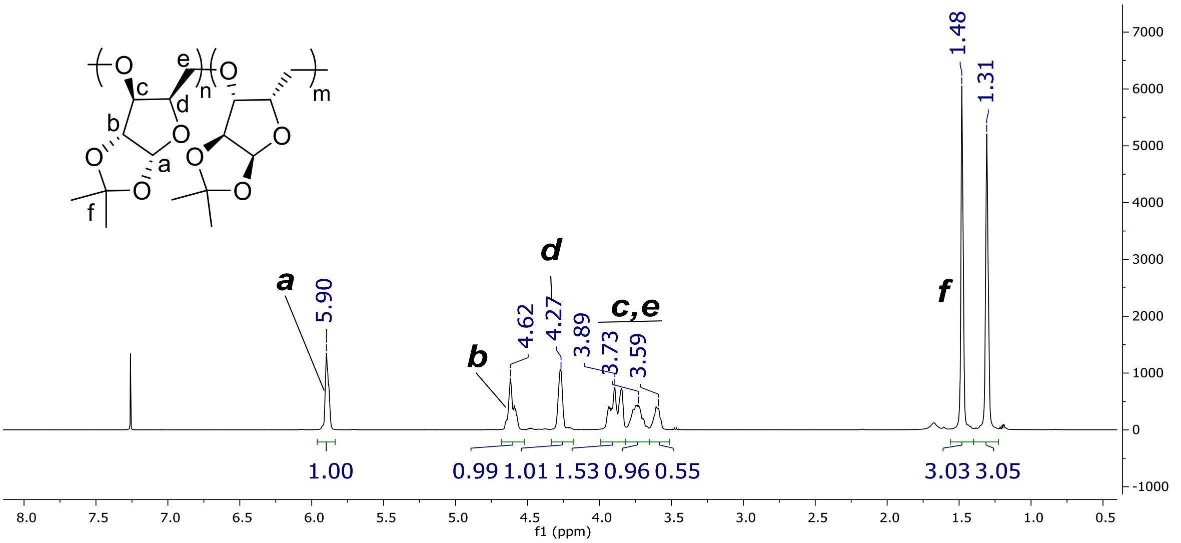

Figure S9.^{1}H NMR spectrum (500 MHz, $CDCl_3$) of 50:50 poly(**D-1-*co*-L-1**) ($CHCl_3$ residual signal at 7.26 ppm).

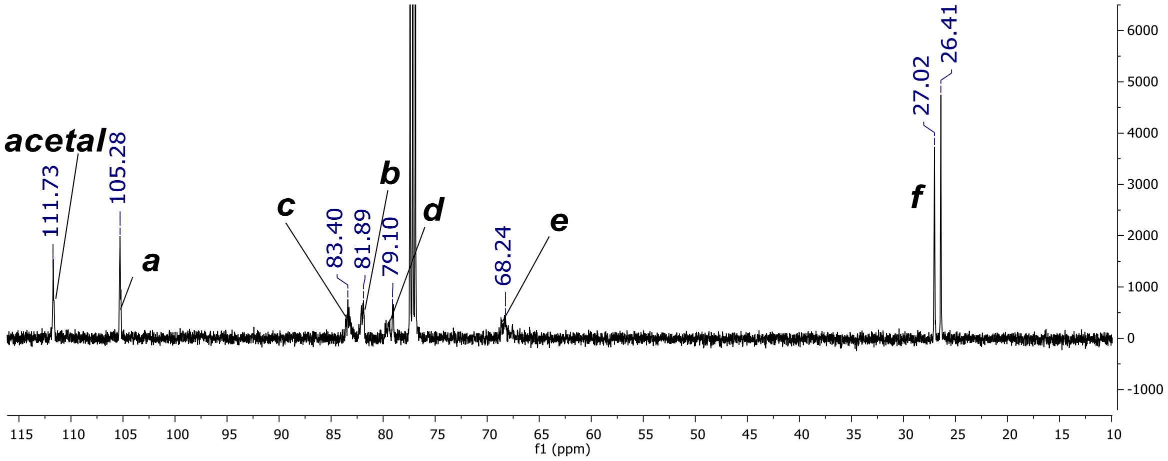

Figure S10.^{13}C{^{1}H} NMR spectrum (126 MHz, $CDCl_3$) of 50:50 poly(**D-1**-*co*-**L-1**) ($CHCl_3$ residual signal at 77.2 ppm).

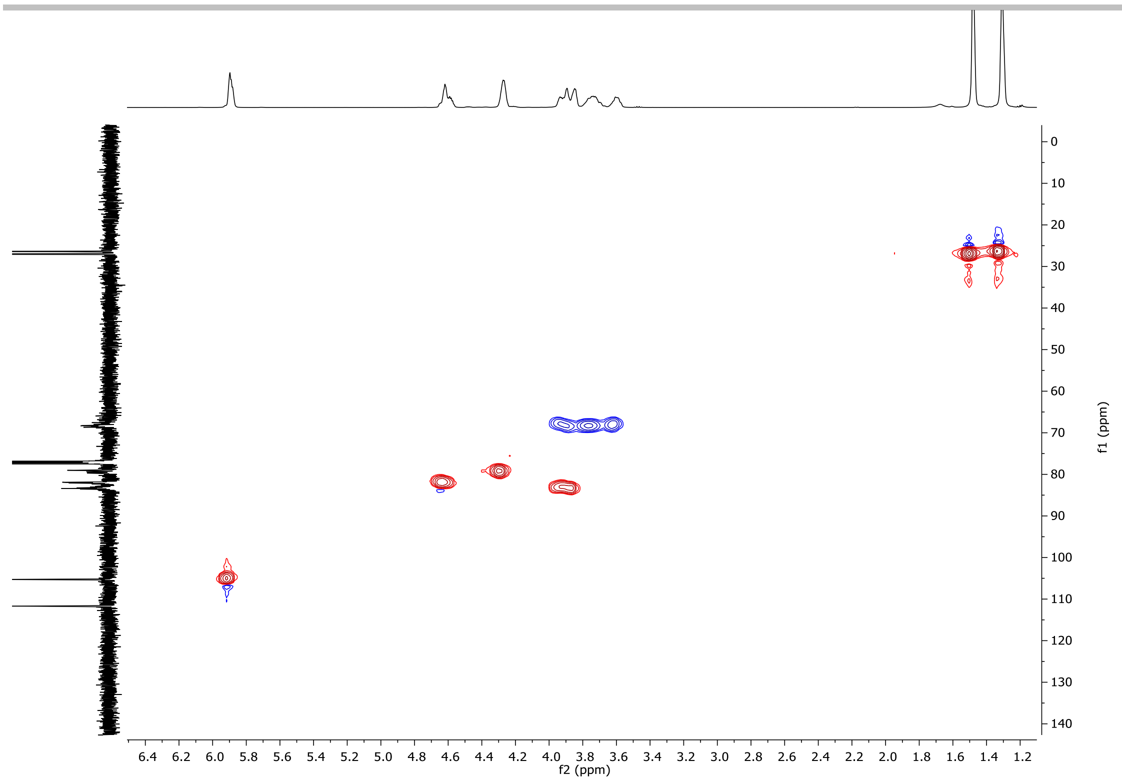

Figure S11.$^{13}C\{^1H\}$ HSQC NMR spectrum ($CDCl_3$) of 50:50 poly(**D-1**-*co*-**L-1**).

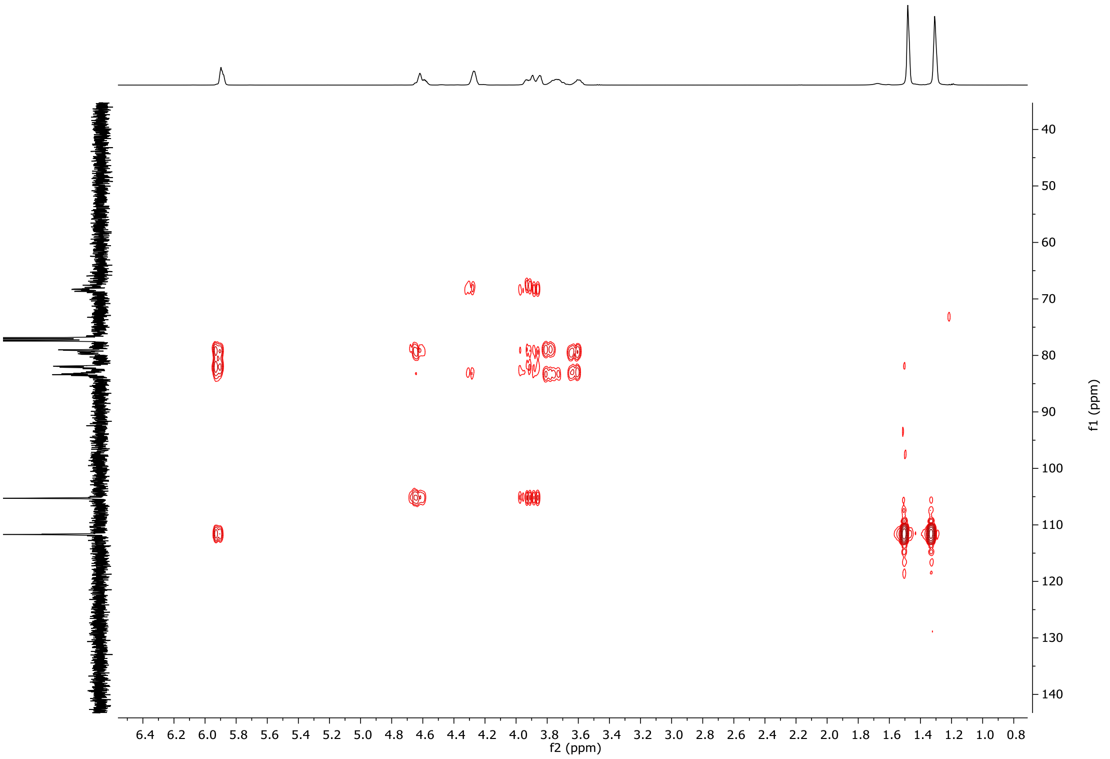

Figure S12.^1H- $^{13}C\{^1H\}$ HMBC NMR spectrum ($CDCl_3$) of 50:50 poly(**D-1**-*co*-**L-1**).

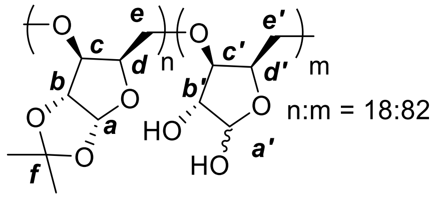

82% deprotected poly (D-1) /poly(L-1) (α:β ~47:53 *N.B. α and β anomers not fully resolved*)

^{1}H NMR (500 MHz, DMSO-d_6) δ 6.34 – 5.66 (m, H_a + **OH**), 5.58 – 5.01 (m, $H_{a'β}$ + **OH**), 4.98 – 4.82 (m, $H_{a'α}$), 4.66 – 4.57 (m, H_b), 4.26 – 4.02 (m, H_d, $H_{d'}$), 4.00 – 3.64 (m, H_c, H_e, $H_{b'}$, $H_{c'}$, $H_{e'}$), 3.62 – 3.47 (m, $H_{e'}$), 3.47 – 3.37 (m, $H_{e'}$), 1.40 (s, H_f), 1.25 (s, H_f). **^{13}C{^{1}H} NMR (126 MHz, DMSO-d_6)** δ 104.6 (C_a), 102.9 ($C_{a'α}$), 96.0 ($C_{a'β}$), 85.8 – 83.4 ($C_{b'}$), 81.2 (C_b), 79.9 – 77.2 ($C_{d'}$, C_d), 76.4 (C_c, $C_{c'}$), 74.2 (C_c, $C_{c'}$), 69.3 (C_e, $C_{e'}$).26.6 (C_f), 26.1 ($C_{f'}$)

M_{nSEC} = 9700; $Đ_M$ = 1.39; T_g *not observed*; T_m = *not observed*; T_c = *not observed*; $T_{d,onset}$ ~ 145 °C.

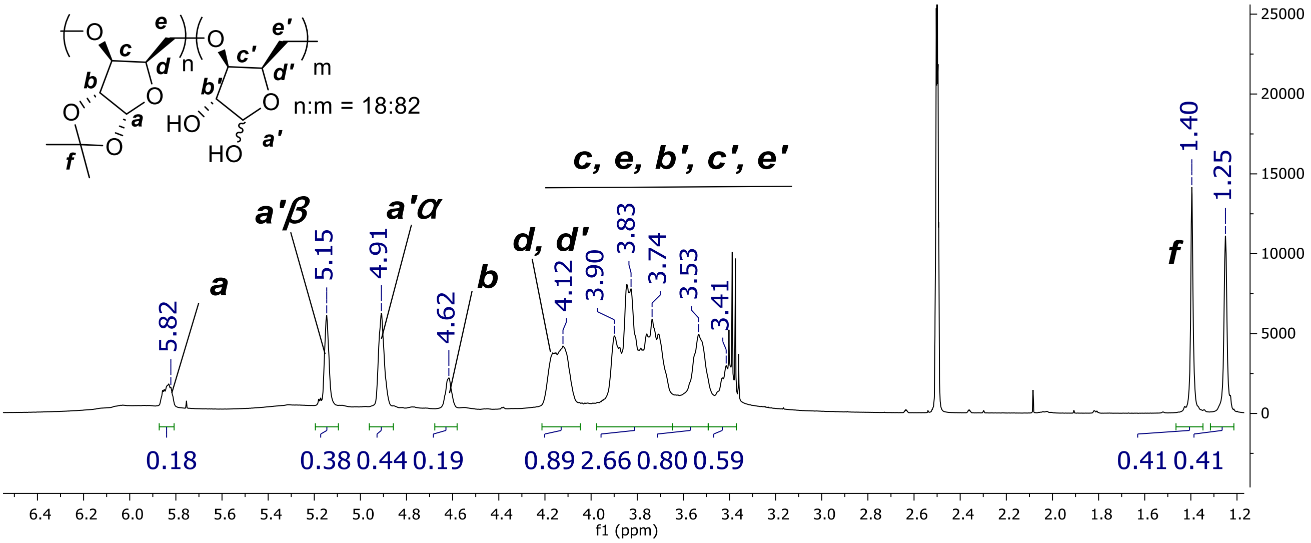

Figure S13. ^{1}H NMR spectrum (500 MHz, d^6-DMSO) of 82% deprotected poly**(L-1**) (DMSO residual signal at 2.50 ppm, Et_2O residual signal at 3.38 ppm).

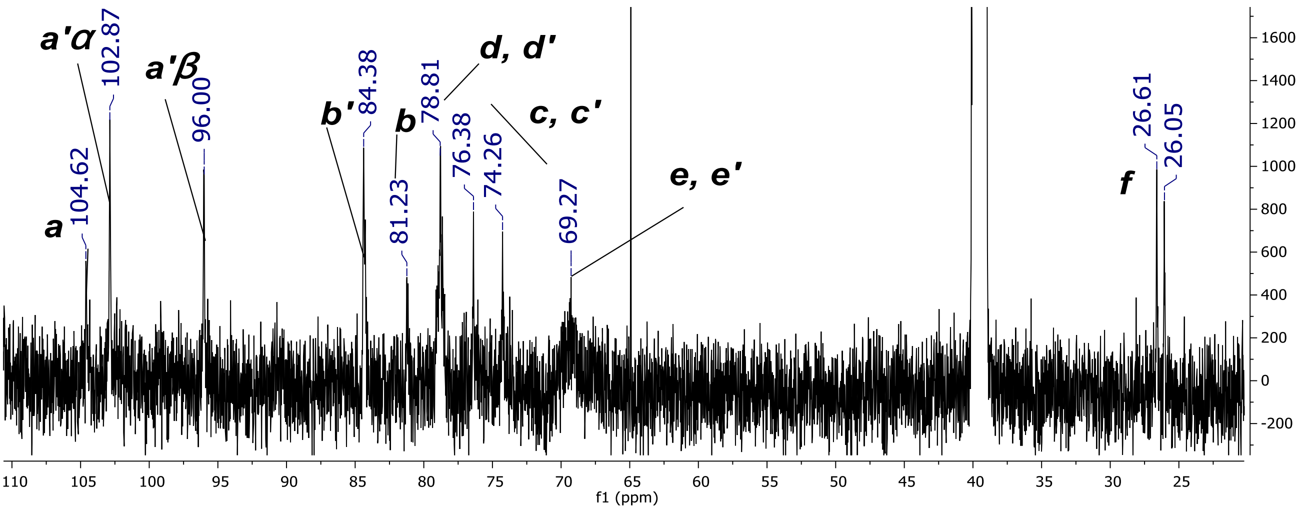

Figure S14. ^{13}C{^{1}H} NMR spectrum (126 MHz, d^6-DMSO) of 82% deprotected poly**(L-1**) (DMSO residual signal at 39.52 ppm, Et_2O residual signal at 64.9 ppm).

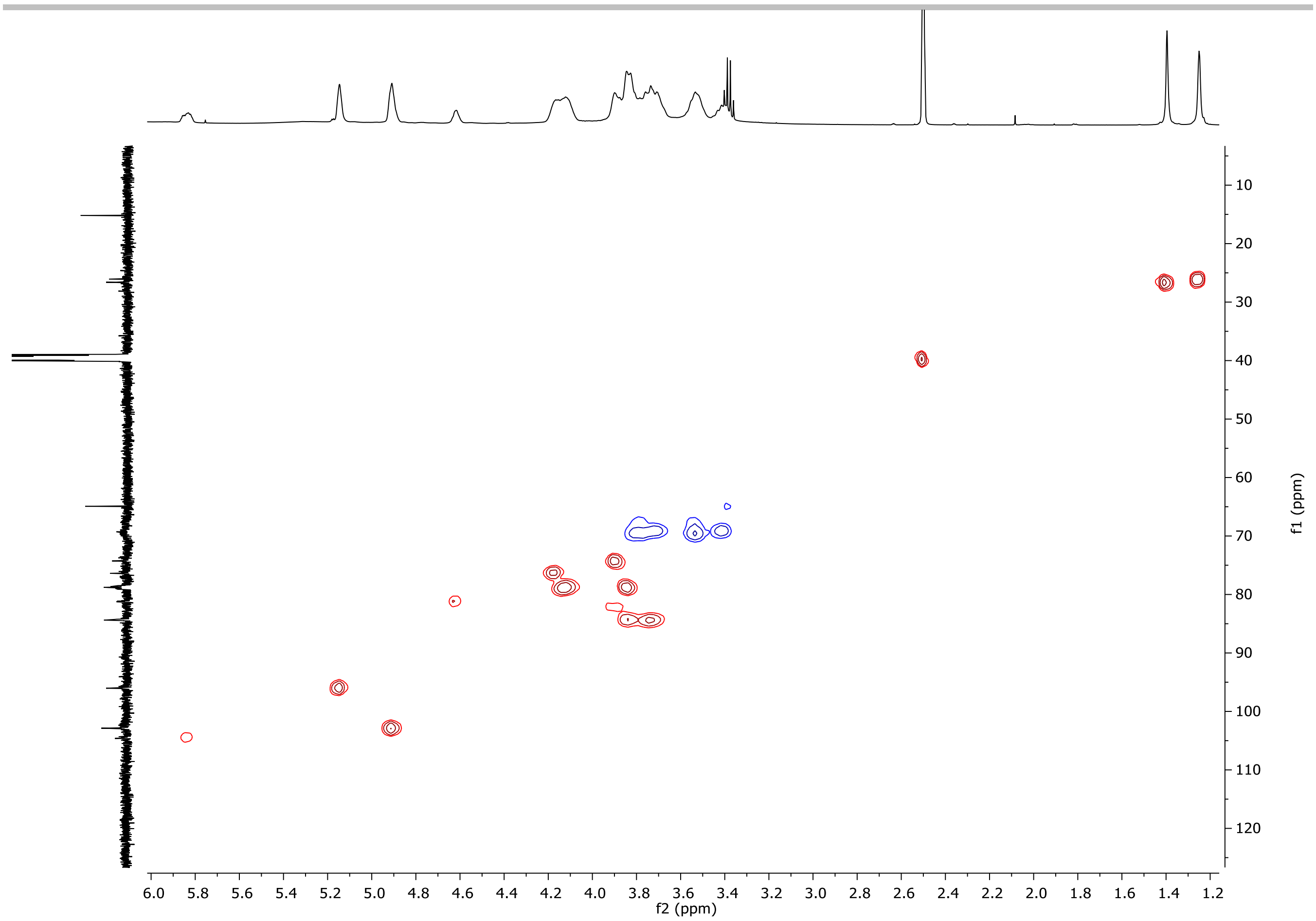

Figure S15.$^{13}C\{^{1}H\}$ HSQC NMR spectrum (d^6-DMSO) of 82% deprotected poly(**L-1**).

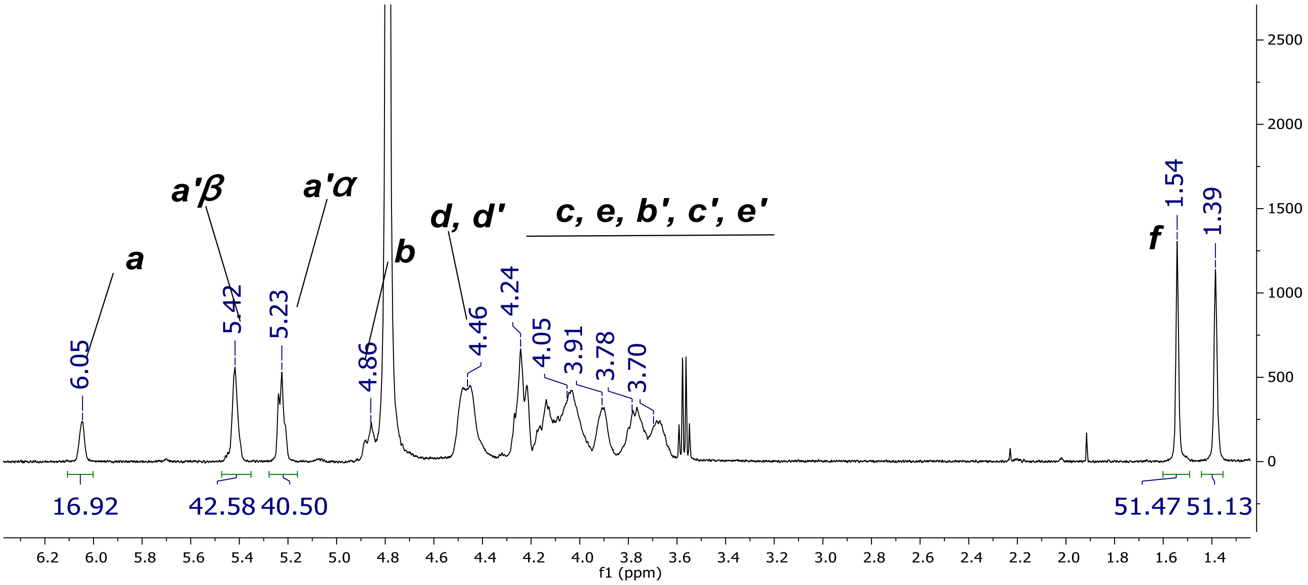

Figure S16. ^{1}H NMR spectrum (500 MHz, D_2O) of 83% deprotected poly(**L-1**) (Et_2O residual signal at 3.56 ppm, H_2O signal at 4.79 ppm).

4. Kinetics

4.1. Polymerization kinetics

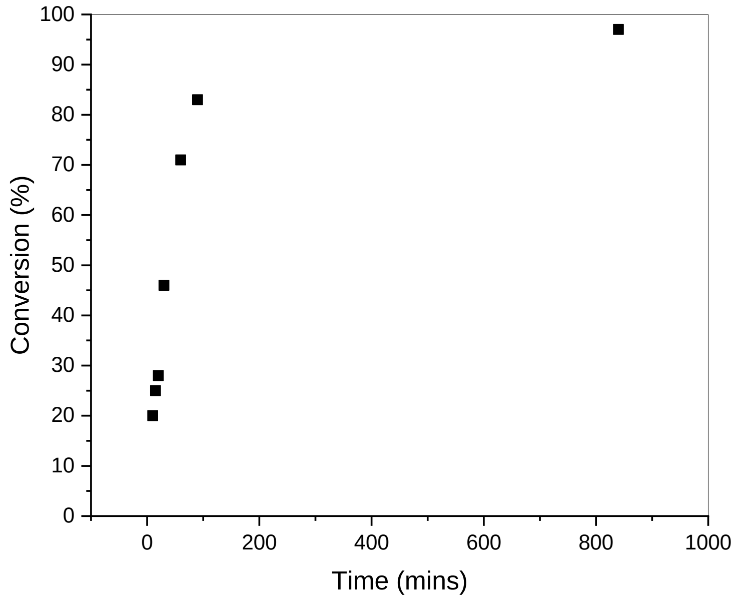

Figure S17. Time *vs* **D-1** conversion (determined by ^{1}H NMR spectroscopy by relative integration of the anomeric protons in **D-1** (δ = 6.27 ppm (d, *J* = 3.7 Hz)) and poly(**D-1**) (δ = 5.88 ppm (d, *J* = 3.5 Hz))) for the KO^{t}Bu initiated ROP of **D-1** at [**D-1**]$_0$:[KO^{t}Bu]$_0$ loadings of 20:1 at 150 °C.

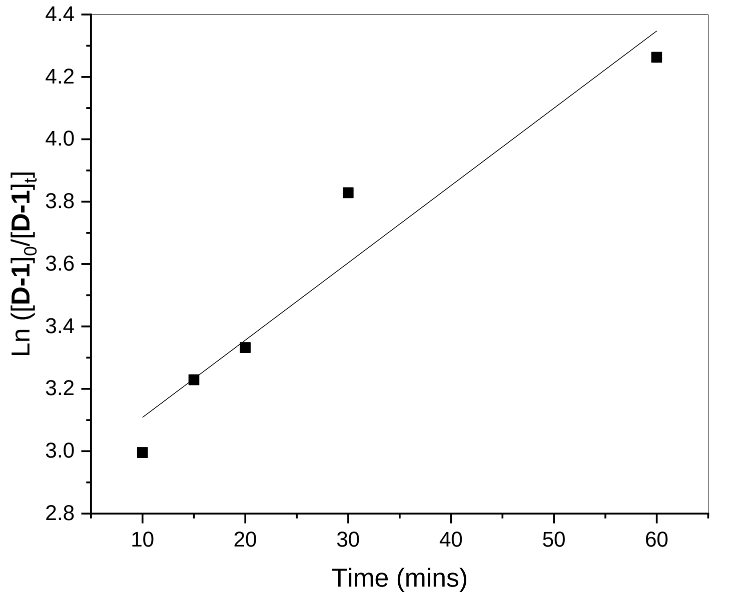

Figure S18. Time *vs* Ln[**D-1**]$_0$/[**D-1**]$_t$ for the KO^{t}Bu initiated ROP of **D-1** at [**D-1**]$_0$:[KO^{t}Bu]$_0$ loadings of 20:1 at 150 °C (R^2 = 0.932, k = 0.0248 min^{-1}). Experiments were performed in batch.

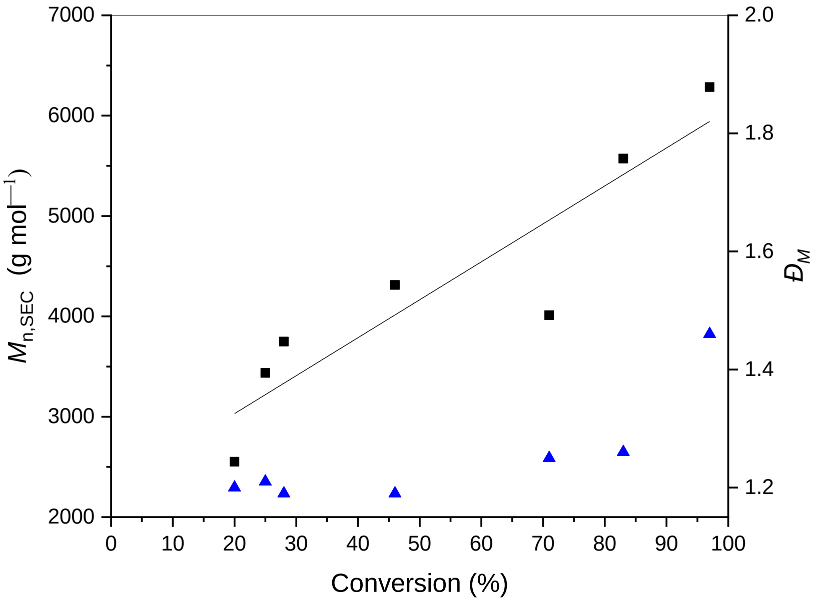

Figure S19. Conversion of **D-1** (determined by ^{1}H NMR spectroscopy by relative integration of the anomeric protons in **D-1** (δ = 6.27 ppm (d, *J* = 3.7 Hz)) and poly(**D-1**) (δ = 5.88 ppm (d, *J* = 3.5 Hz)) *vs* $M_{n,SEC}$ (black squares) and $Đ_M$ (red crosses) for the KO^{t}Bu initiated ROP of **D-1** at [**D-1**]$_0$:[KO^{t}Bu]$_0$ loadings of 20:1 at 150 °C (R^2 = 0.838). Experiments were performed in batch (black square = $M_{n,SEC}$, blue triangle = $Đ_M$).

4.2. Deprotection kinetics

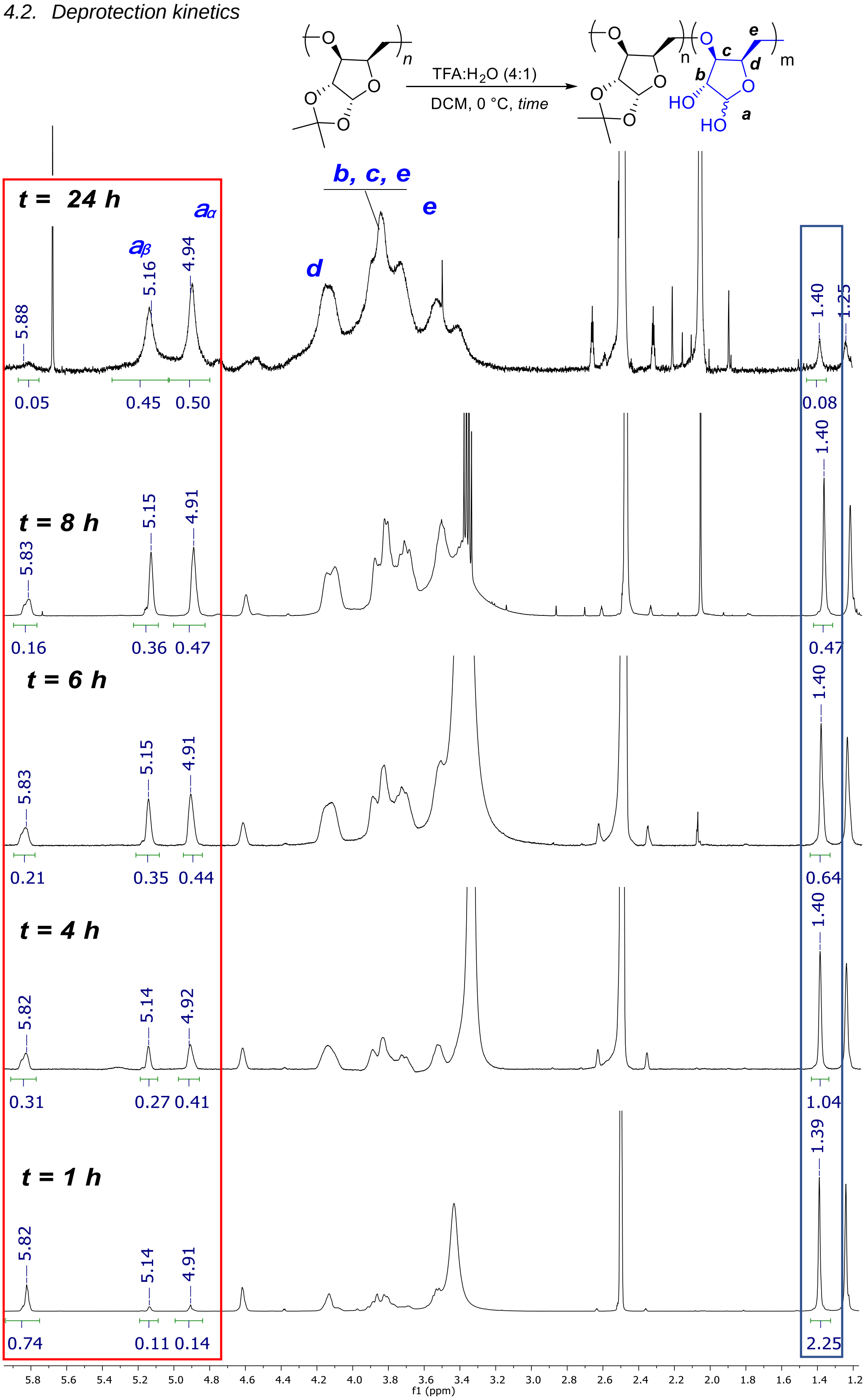

Figure S20. Representative ^{1}H NMR spectra (400 MHz, d^{6}-DMSO, 1.30–6.20 ppm) of aliquots taken during deacetalization of poly(**D-1**) and poly (**L-1**). Reaction performed at 0 °C with 80% aq TFA in DCM. $[TFA]_0$ = 7.5 M. Conversion determined by ^{1}H NMR spectroscopy by relative integration of the anomeric protons (1H, highlighted in red box, δ = 4.97 ppm (b), δ = 5.14 ppm (b) and δ = 5.83 ppm) and methyl acetal group (3H, highlighted in blue box, δ = 1.40 ppm (s)).

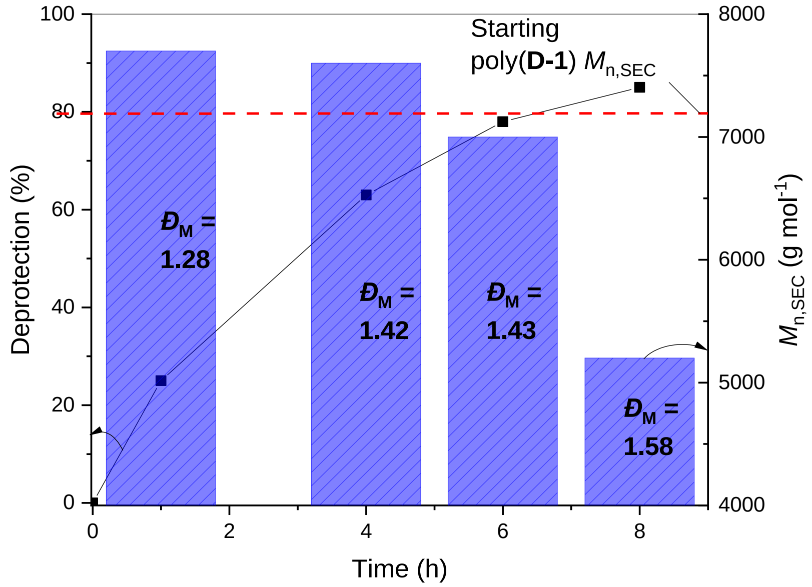

Figure S21. Time *vs* % deprotection of poly(**D-1**) (determined by ^{1}H NMR spectroscopy, see Fig. S16) *vs* $M_{n,SEC}$ for deprotected poly(**D-1**). $M_{n,SEC}$ values taken from samples heated prior to SEC analysis. Reaction performed at 0 °C with 80% aq TFA in DCM. $[TFA]_0$ = 7.5 mol L^{-1}. (black line = % deprotection, blue bars = $M_{n,SEC}$ and red dashed line = $M_{n,SEC}$ of poly(**D-1**) used in deprotection).

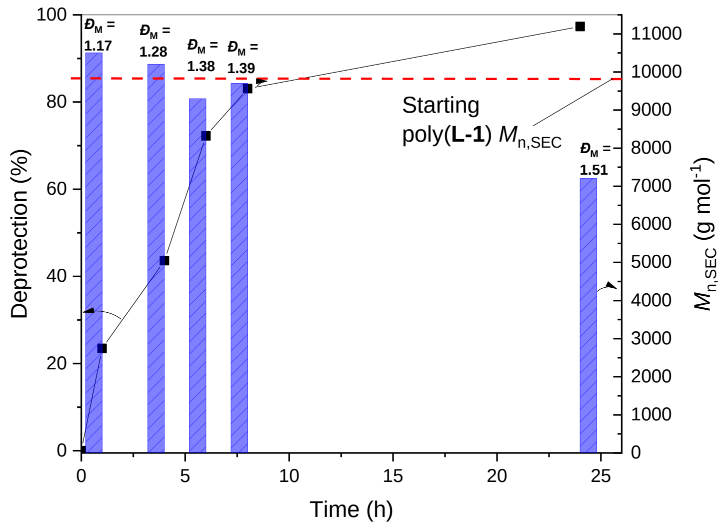

Figure S22. Time *vs* % deprotection of poly(**L-1**) (determined by ^{1}H NMR spectroscopy, see Fig. S16) *vs* $M_{n,SEC}$ for deprotected poly(**L-1**). $M_{n,SEC}$ values taken from samples heated prior to SEC analysis. Reaction performed at 0 °C with 80% aq TFA in DCM. $[TFA]_0$ = 7.5 mol L^{-1}. (black line = % deprotection, blue bars = $M_{n,SEC}$ and red dashed line = $M_{n,SEC}$ of poly(**L-1**) used in deprotection).

5. Additional polymerization results

Reactions with commonly used ROP catalysts $Sn(Oct)_2$ and TBD, in combination with an alcohol initiator, both failed to yield polymer, including at 180 °C (Table S1).

Table S1. Polymerization of **D-1** with $Sn(Oct)_2$ and TBD

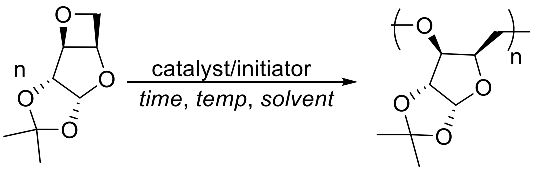

D-1

Entry	Initiator	[**D-1**]$_0$:[I]$_0$	Temp (°C)	Time (h)	Conv (%)[a]	$M_{n,theo}$ (g mol^{-1})	$M_{n,SEC}$[b] (g mol^{-1})
S1[c]	4-MeBnOH/$Sn(Oct)_2$	200:1	120	22	1	-	-
S2[c]	4-MeBnOH/$Sn(Oct)_2$	200:1	180	22	1	-	-
S3[d]	4-MeBnOH/TBD	100:1	120	22	1	-	-
S4[d]	4-MeBnOH/TBD	100:1	180	22	18	3200[e]	<1000 (-)

Reactions carried out in neat **D-1**.[a] Calculated by ^{1}H NMR spectroscopy using the relative integration of the anomeric protons in **D-1** (δ = 6.27 ppm (d, *J* = 3.7 Hz)) and poly(**D-1**) (δ = 5.88 ppm (d, *J* = 3.5 Hz)).[b] Calculated by SEC relative to a polystyrene standard using a THF eluent.[c] [$SnOct_2$]$_0$:[4-MeBnOH]$_0$ = 1:2.[d] [TBD]$_0$:[4-MeBnOH]$_0$ = 1:1.[e] Calculated as M_r(4-MeBnOH) + (M_r (**D-1**) × [**D-1**]$_0$/[4-MeBnOH]$_0$ × conv/ 100%).

6. $^1H\{^1H\}$ NMR spectroscopy

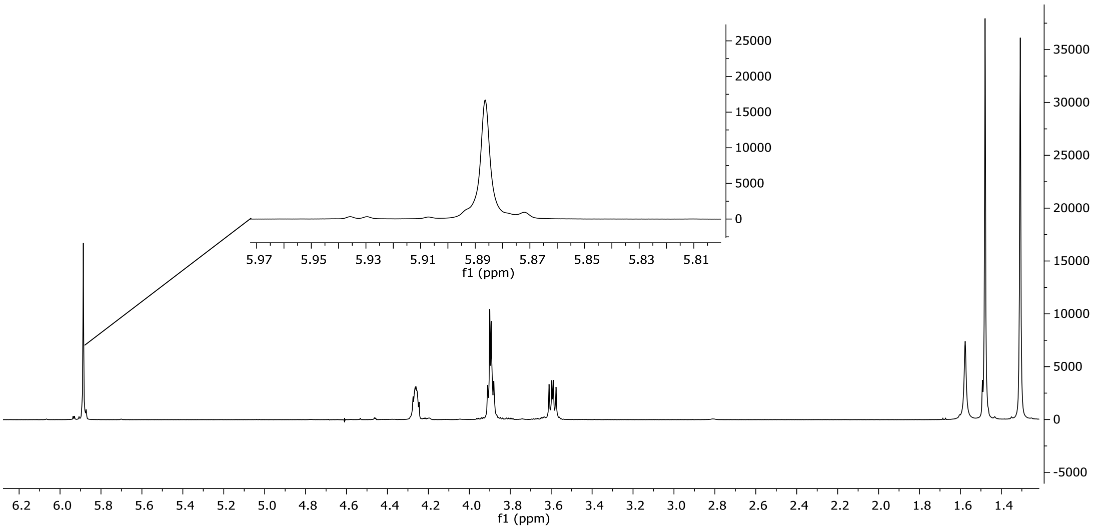

Figure S23. $^1H\{^1H\}$ NMR spectrum (500 MHz, $CDCl_3$) of poly(**D-1**) ($M_{n,SEC}$ = 9200, $Đ_M$ = 1.15, Table 1, entry 5) irradiated at δ = 4.62 ppm.

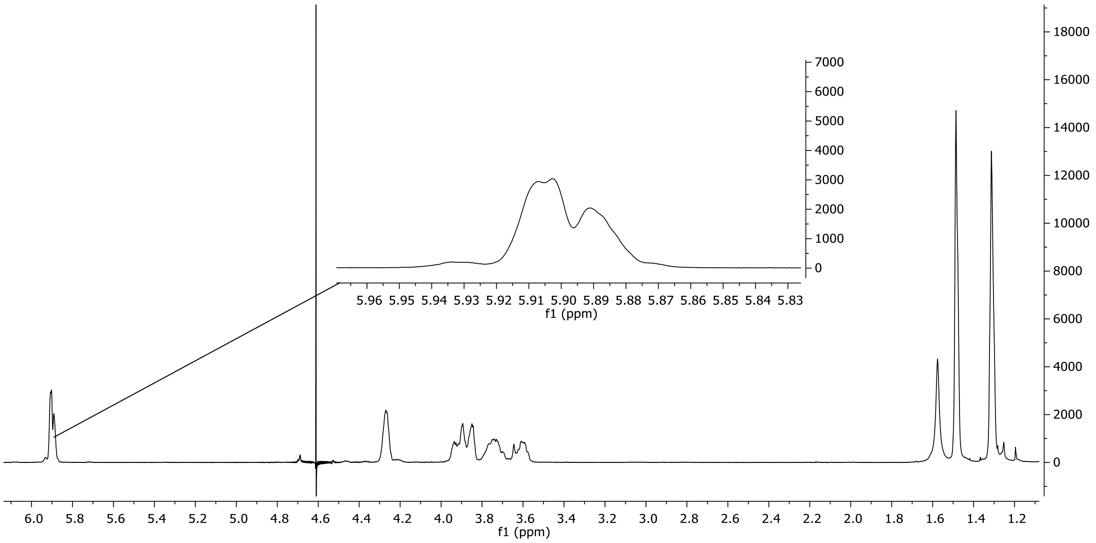

Figure S24. $^1H\{^1H\}$ NMR spectrum (500 MHz, $CDCl_3$) 50:50 poly (**D-1**-*co*-**L-1**) ($M_{n,SEC}$ = 9700, $Đ_M$ = 1.18, Table 1, entry 14) irradiated at δ = 4.62 ppm.

7. SEC chromatograms

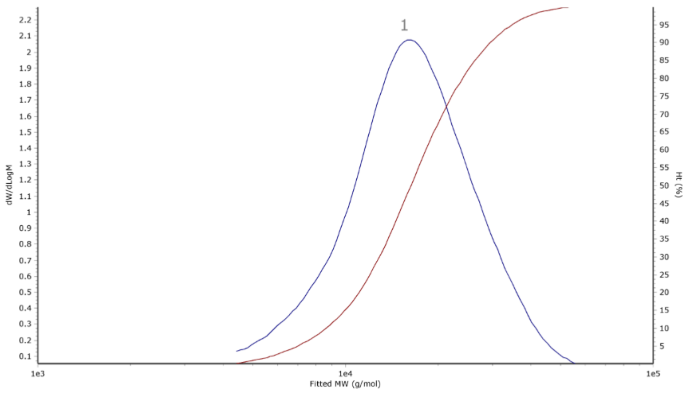

Figure S25. SEC chromatogram of poly(**D-1**) ($M_{n,SEC}$ = 14500, $Đ_M$ = 1.25) measured in THF.

.

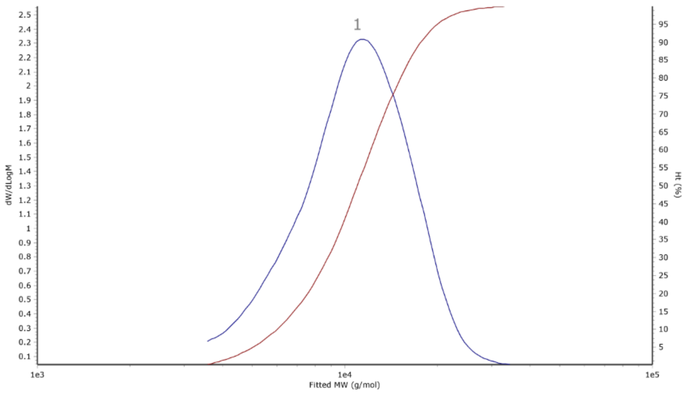

Figure S26. SEC chromatogram of poly(**L-1**) ($M_{n,SEC}$ = 9800, $Đ_M$ = 1.19) measured in THF.

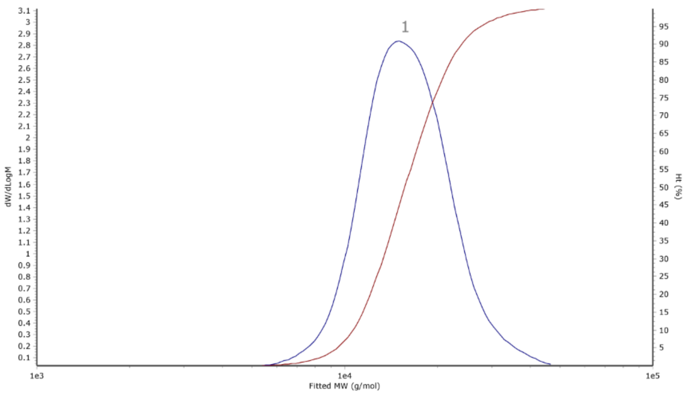

Figure S27.SEC chromatogram of poly(**D-1**-*co*-**L-1**) ($M_{n,SEC}$ = 15000, $Đ_M$ = 1.11) measured in THF.

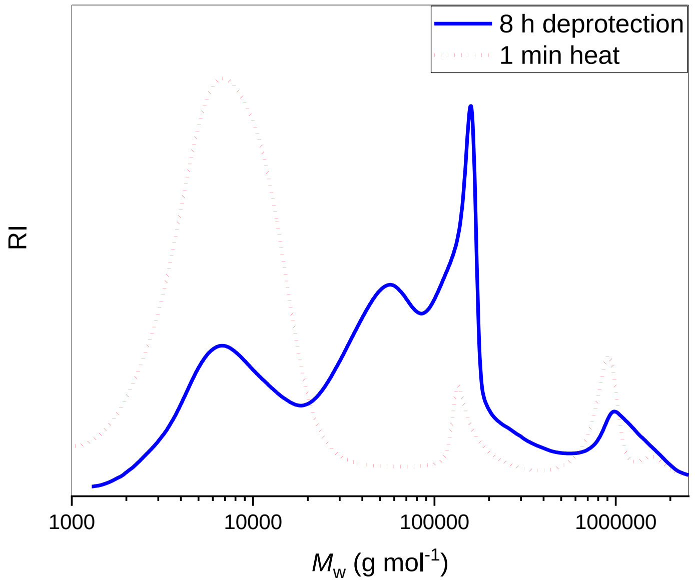

Figure S28. SEC chromatograms of poly(**D-1**) deprotected at 85% before (blue) and after heating (red, dashed) measured in DMF.

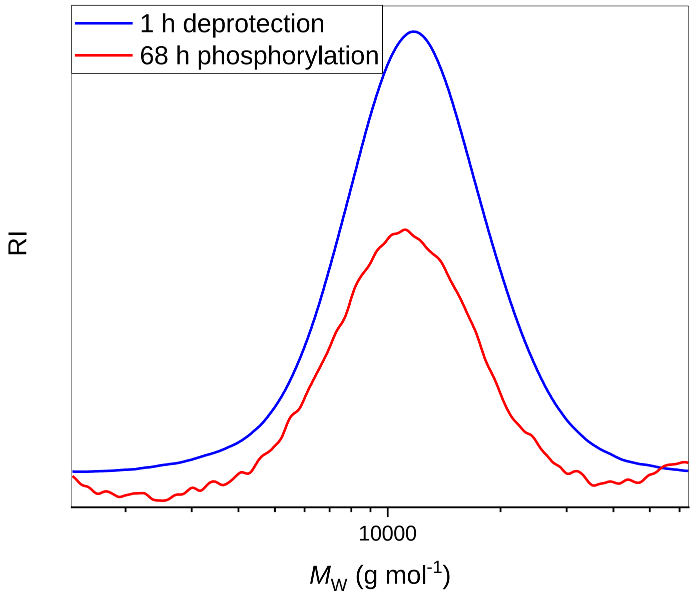

Figure S29. SEC chromatograms of poly(**D-1**) deprotected at 25% (blue, $M_{n,SEC}$ = 10700 g mol^{-1}, $Đ_M$ = 1.23) and phosphorylated polymer (red, $M_{n,SEC}$ = 10100 g mol^{-1}, $Đ_M$ = 1.19) measured in DMF.

8. End group analysis

Table S2. MALDI-ToF spectrum of OEt end-capped poly(**D-1**) ($M_{n,SEC}$ = 4000 g mol^{-1}, $M_{n,NMR}$ = 4040 g mol^{-1} and $M_{n,MALDI}$ = 4060 g mol^{-1}, Table 1, entry 8).

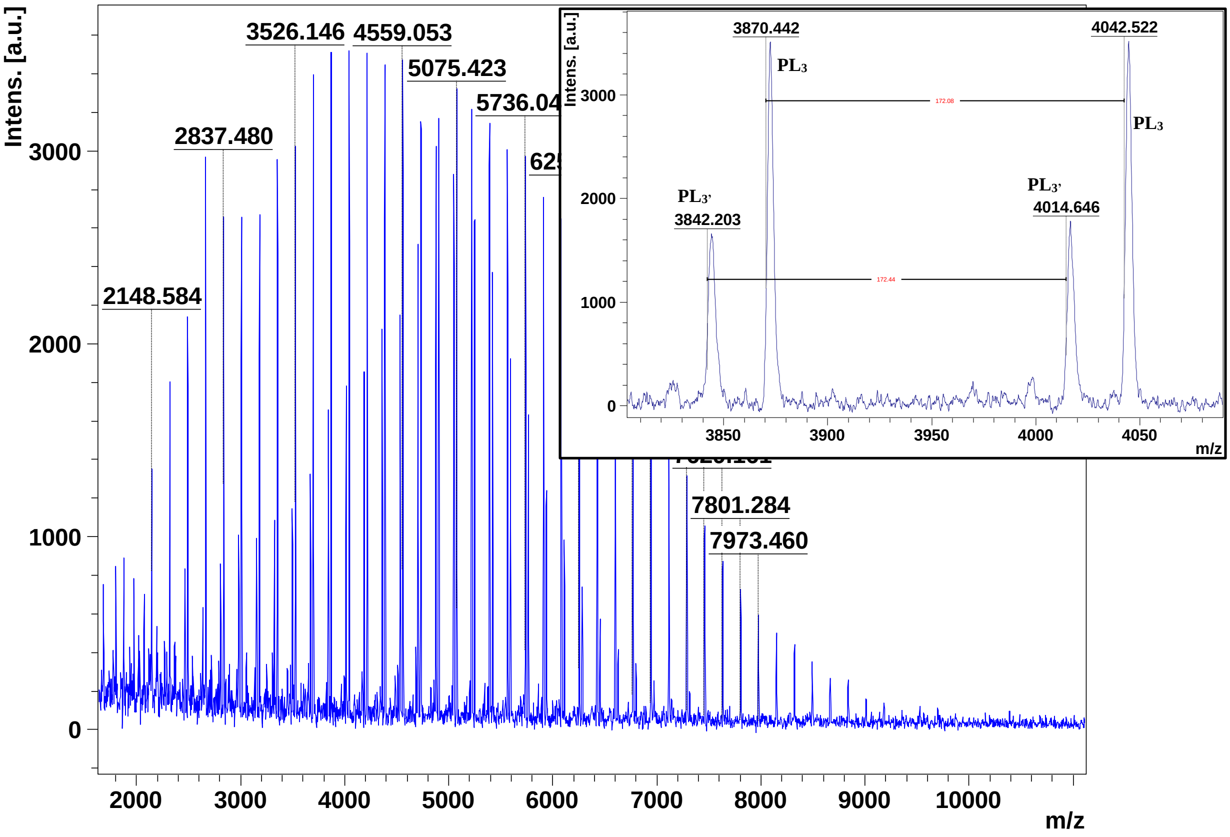

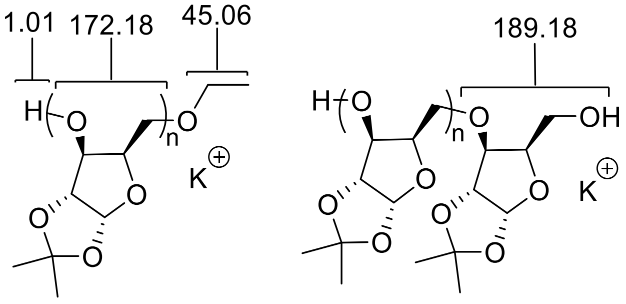

Primary series: **PL$_3$ + K$^+$** Secondary series: **PL$_{3'}$ + K$^+$**

Structure	n	m/z_{theo} (g mol^{-1})[a]	m/z_{MALDI}(g mol^{-1})
P_{L3}+ K$^+$	22	3873	3870
$P_{L3'}$+ K$^+$	21	3845	3842

[a] Calculated as M_r (I) +M_r(K)+ (M_r (D-1) × n).

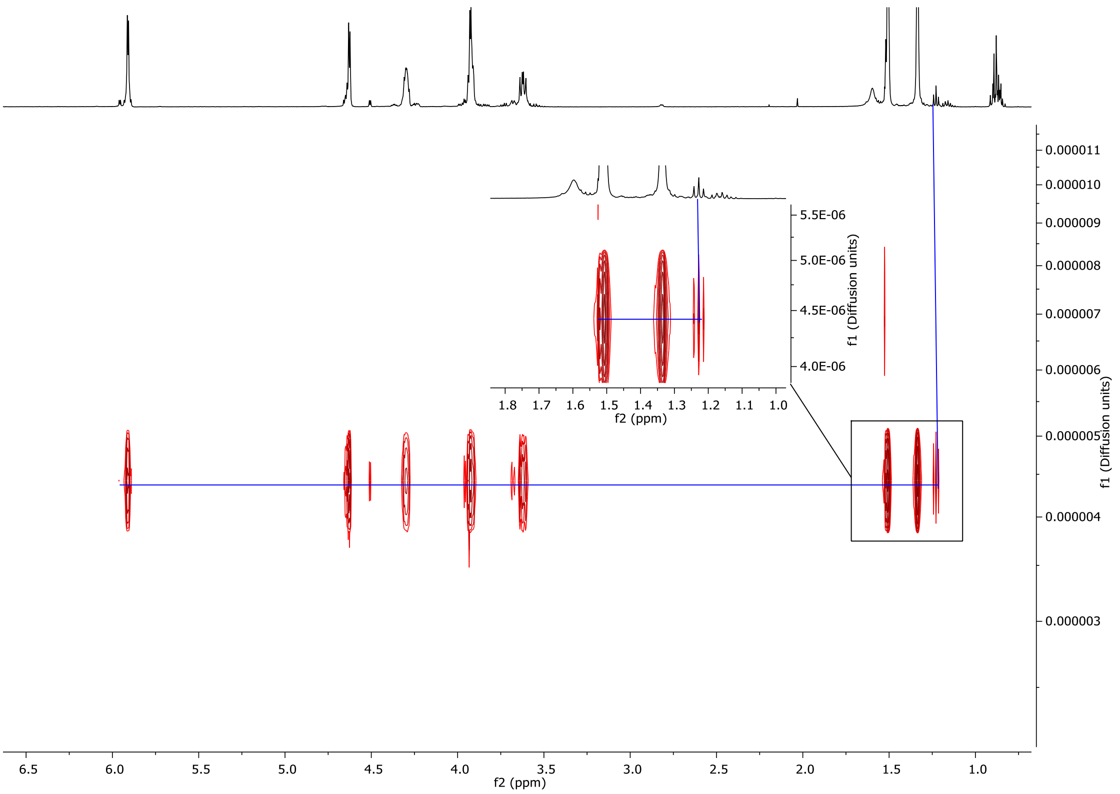

Figure S30. ^{1}H DOSY NMR spectrum (500 MHz, $CDCl_3$) of OEt-end capped poly(**D-1**) ($M_{n,SEC}$ = 4000 g mol^{-1}, $M_{n,NMR}$ = 4040 g mol^{-1} and $M_{n,MALDI}$ = 4060 g mol^{-1}).

9. Thermal characterization

9.1. TGA traces

9.1.1. Homochiral and heterochiral polymers

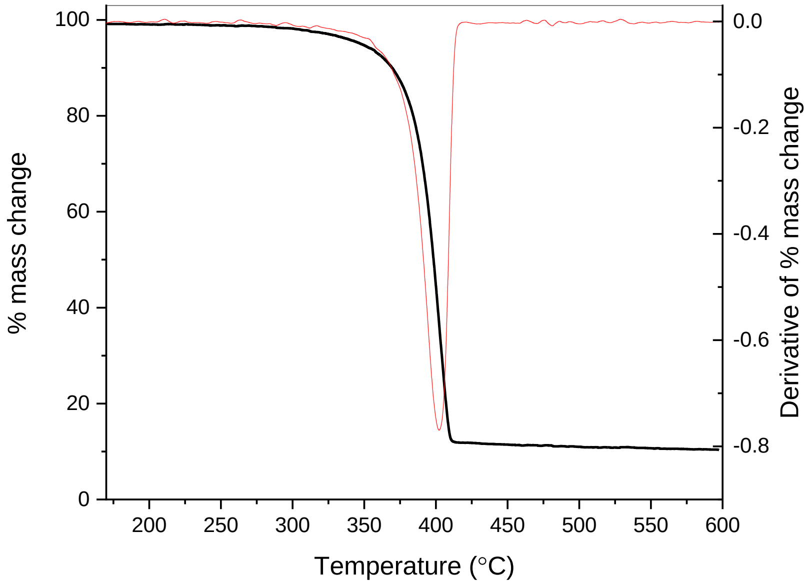

Figure S31. TGA trace of poly(**D-1**) ($M_{n,SEC}$ = 9200 g mol^{-1}, $Đ_M$ = 1.15, Table 1, entry 5). Obtained values: $T_{d,onset}$ = 315 °C; T_{d5} = 348 °C; T_{max}= 402 °C with 13% char remaining at 600 °C.

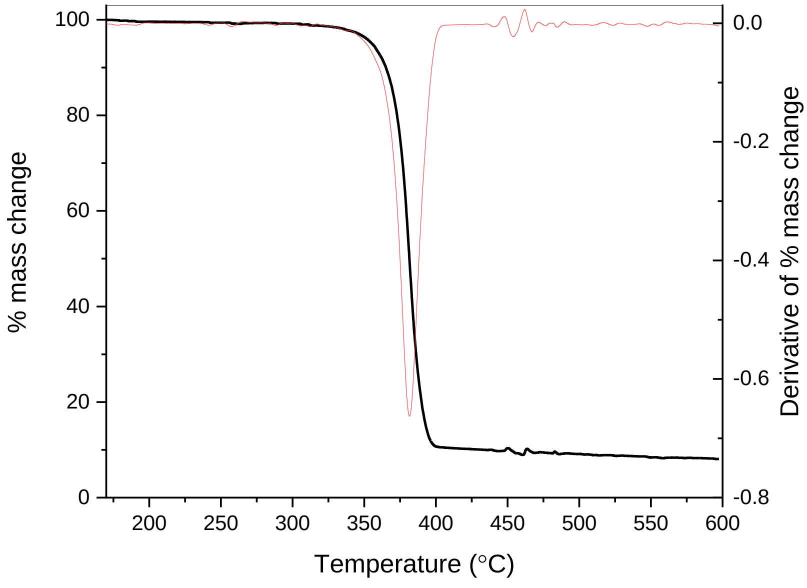

Figure S32. TGA trace of poly(**L-1**) ($M_{n,SEC}$ = 9800 g mol^{-1}, $Đ_M$ = 1.19, Table 1, entry 11). Obtained values: $T_{d,onset}$ = 318 °C; T_{d5} = 355 °C; $T_{d,max}$= 382 °C with 13% char remaining at 600 °C.

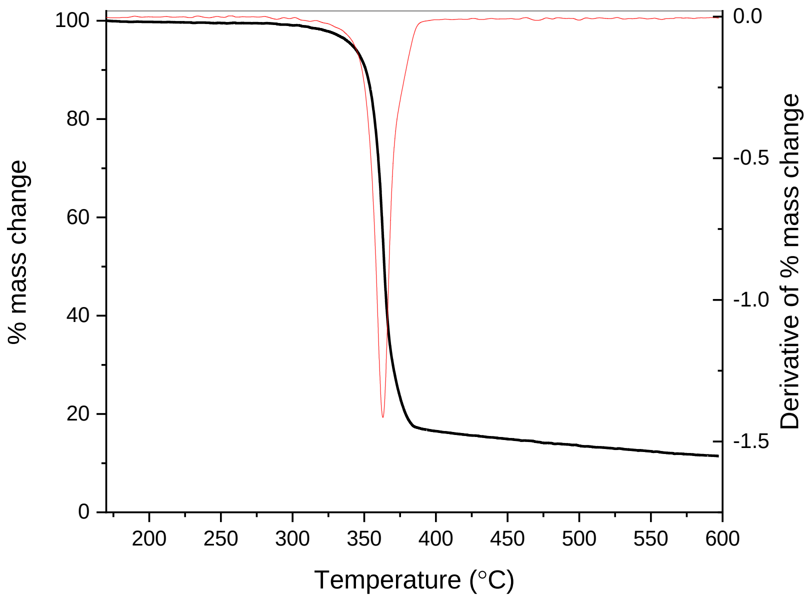

Figure S33. TGA trace of 50:50 poly(**D-1**-*co*-**L-1**) ($M_{n,SEC}$ = 9700 g mol^{-1}, $Đ_M$ = 1.18, Table 1, entry 14). Obtained values: $T_{d,onset}$ = 301 °C; T_{d5} = 342 °C; $T_{d,max}$= 363 °C with 20% char remaining at 600 °C.

9.1.2. Blends

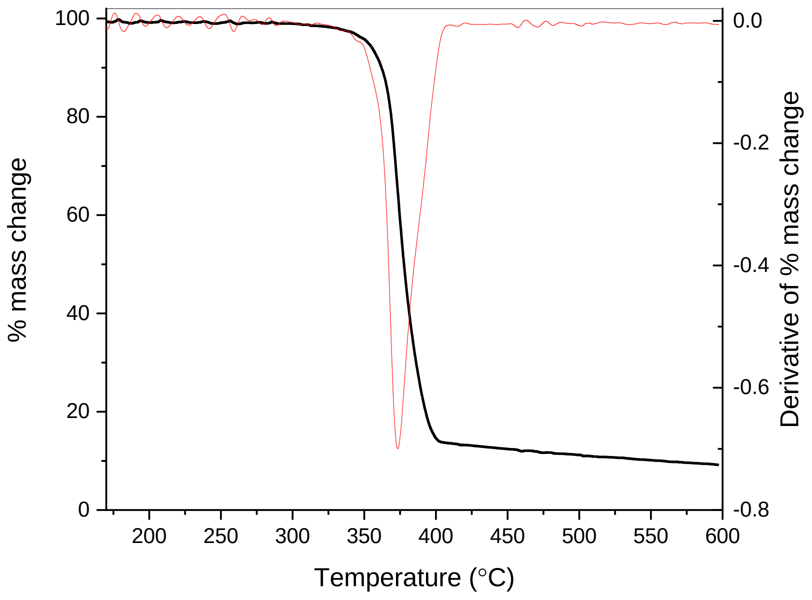

Figure S34. TGA trace of 50:50 blend of poly(**D-1**) and poly(**L-1**). Obtained values: $T_{d,onset}$ = 320 °C; T_{d5} = 353 °C; $T_{d,max}$= 372 °C with 11% char remaining at 600 °C.

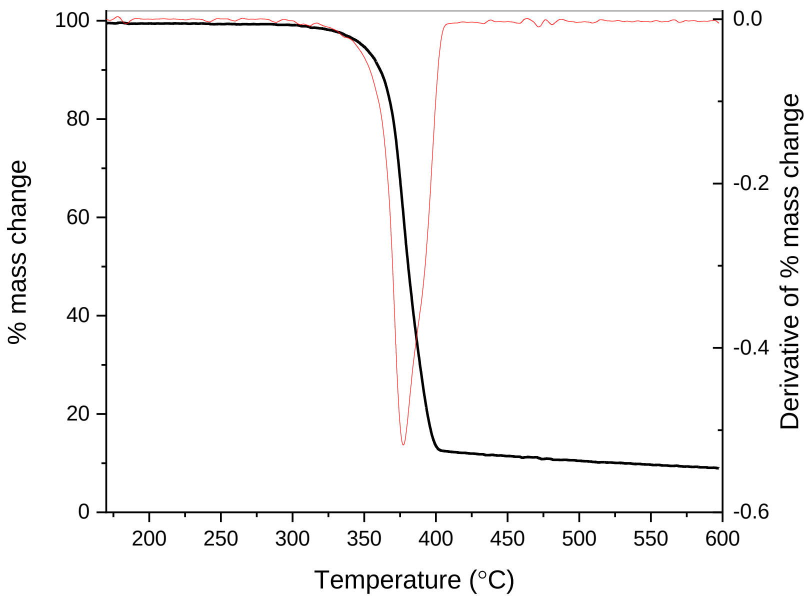

Figure S35. TGA trace of 75:25 blend of poly(**D-1**) and poly(**L-1**). Obtained values: $T_{d,onset}$ = 316 °C; T_{d5} = 349 °C; $T_{d,max}$= 378 °C with 11% char remaining at 600 °C.

9.1.3. *Deprotected polymer*

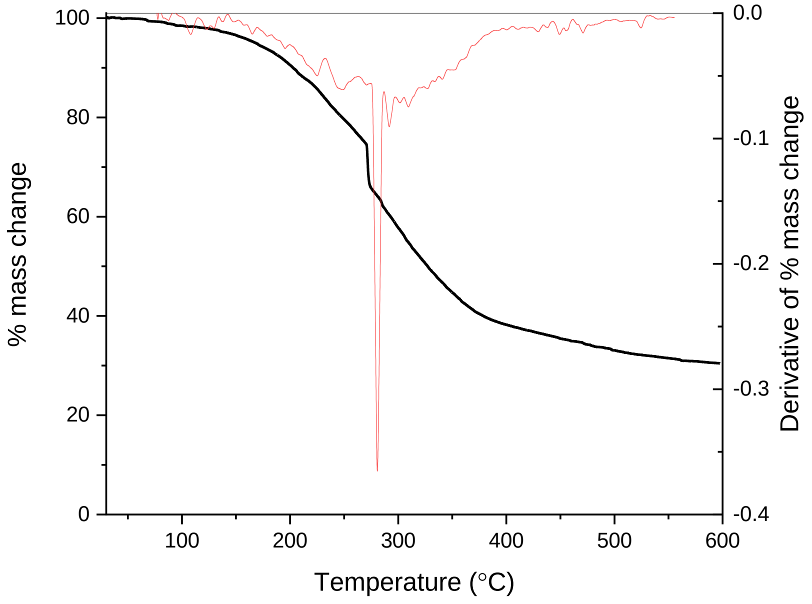

Figure S36. TGA trace of 85% deprotected poly(**D-1**). Obtained values: $T_{d,onset}$ ~145 °C; T_{d5} = 168 °C; $T_{d,max}$= 272 °C with 35% char remaining at 600 °C.

9.2. DSC traces

9.2.1. Homochiral polymers

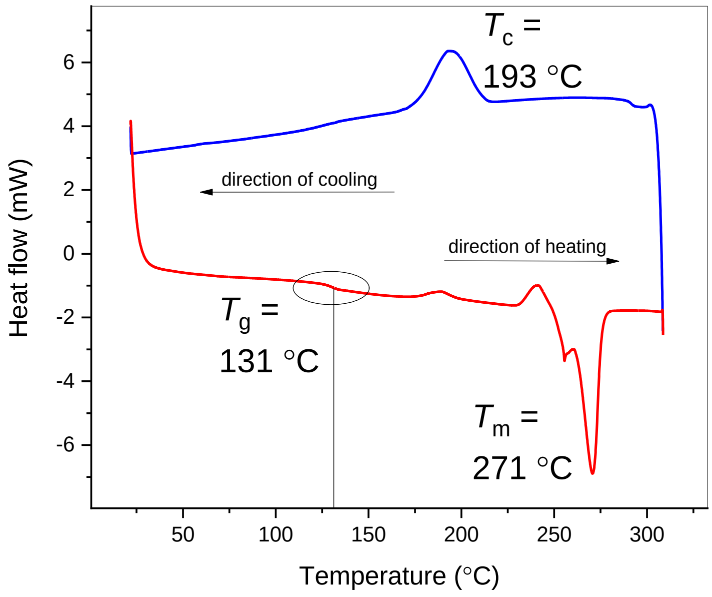

Figure S37. DSC trace collected at 20 °C min^{-1} showing the first cooling and second heating cycle under argon of poly(**L-1**) precipitated from Et2O ($M_{n,SEC}$ = 9800 g mol^{-1}, $Đ_M$ = 1.19; T_g = 131 °C; T_m = 271 °C; T_c = 193 °C, Table 1, entry 11).

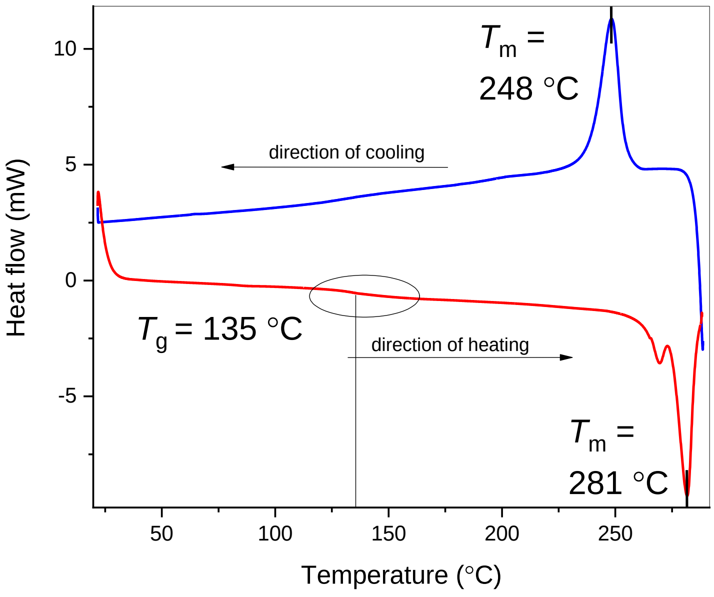

Figure S38. DSC trace collected at 20 °C min^{-1} showing the first cooling and second heating cycle under argon of poly(**D-1**) precipitated from Et_2O ($M_{n,SEC}$ = 9200 g mol^{-1}, $Đ_M$ = 1.15; T_g = 135 °C; T_m = 248 °C; T_c = 248 °C, Table 1, entry 5).

9.2.2. *Heterochiral polymers*

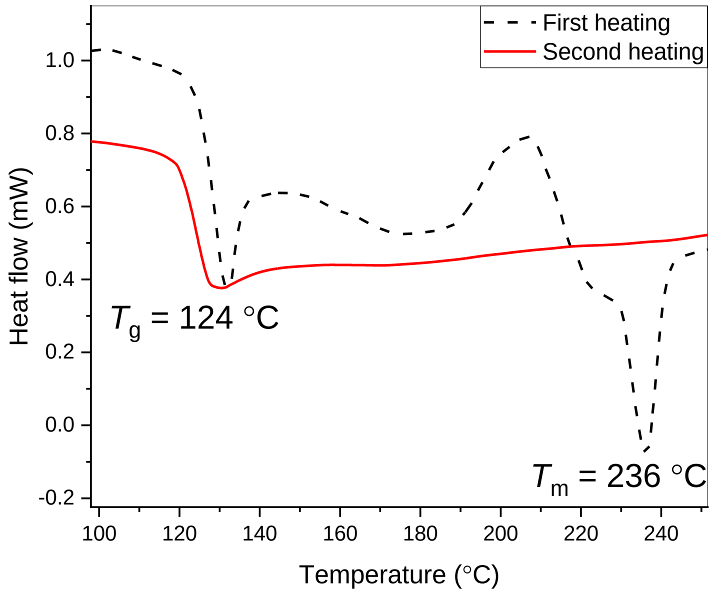

Figure S39. DSC trace collected at 20 °C min^{-1} showing the first and second heating cycle under argon of 90:10 poly(**D-1**-*co*-**L-1**) precipitated from hexane ($M_{n,SEC}$ = 7900 g mol^{-1}, $Đ_M$ = 1.20; T_g = 124 °C; T_m = not observed; T_c = not observed, Table 1, entry 12).

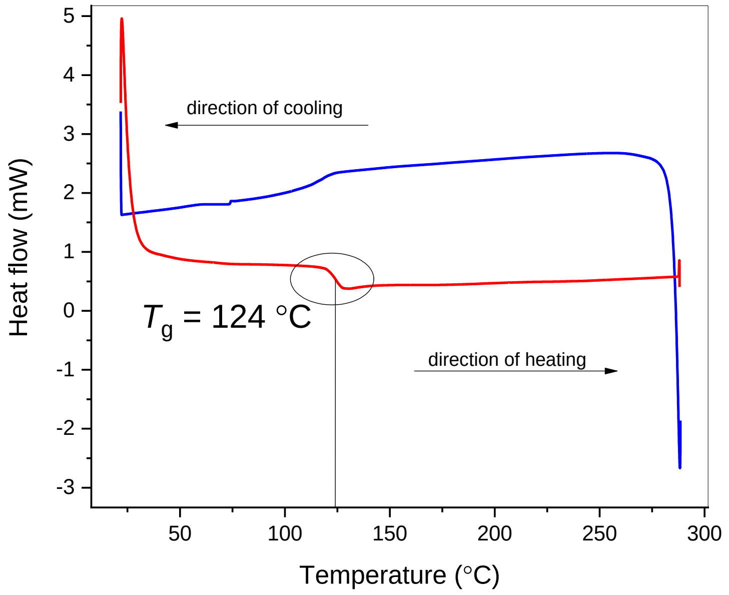

Figure S40. DSC trace collected at 20 °C min^{-1} showing the first cooling and second heating cycle under argon of 90:10 poly(**D-1**-*co*-**L-1**) precipitated from hexane ($M_{n,SEC}$ = 7900 g mol^{-1}, $Đ_M$ = 1.20; T_g = 124 °C; T_m = not observed; T_c = not observed, Table 1, entry 12).

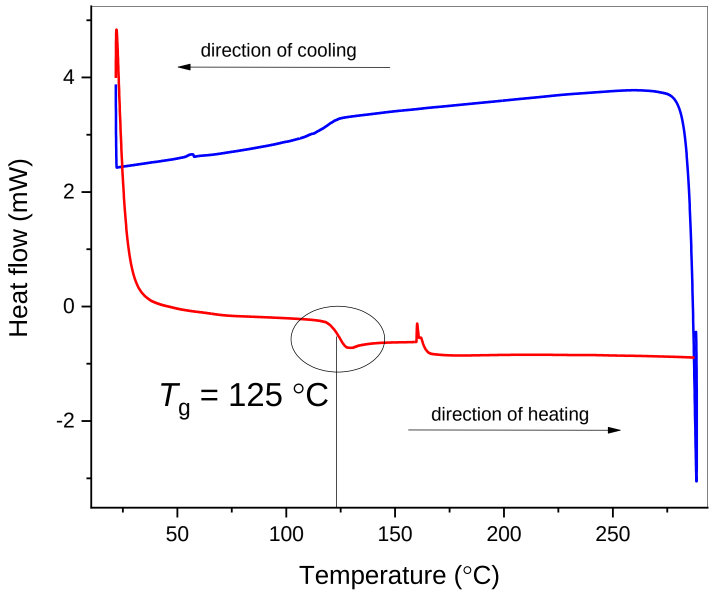

Figure S41. DSC trace collected at 20 °C min^{-1} showing the first cooling and second heating cycle under argon of 70:30 poly(**D-1**-*co*-**L-1**) precipitated from hexane ($M_{n,SEC}$ = 10100 g mol^{-1}, $Đ_M$ = 1.18; T_g = 125 °C; T_m = not observed; T_c = not observed, Table 1, entry 13).

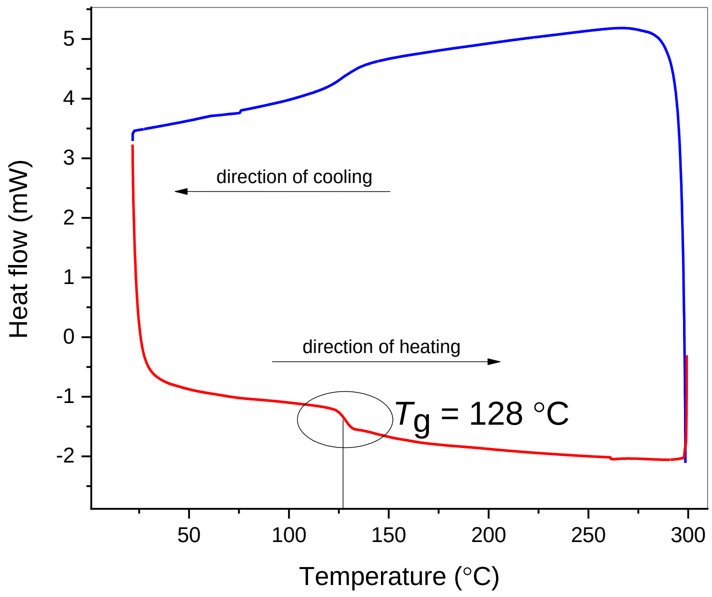

Figure S42. DSC trace collected at 20 °C min^{-1} showing the first cooling and second heating cycle under argon of 50:50 poly(**D-1**-*co*-**L-1**) precipitated from hexane ($M_{n,SEC}$ = 9300 g mol^{-1}, $Đ_M$ = 1.17; T_g = 128 °C; T_m = not observed; T_c = not observed, Table 1, entry 14).

9.2.3. Blends

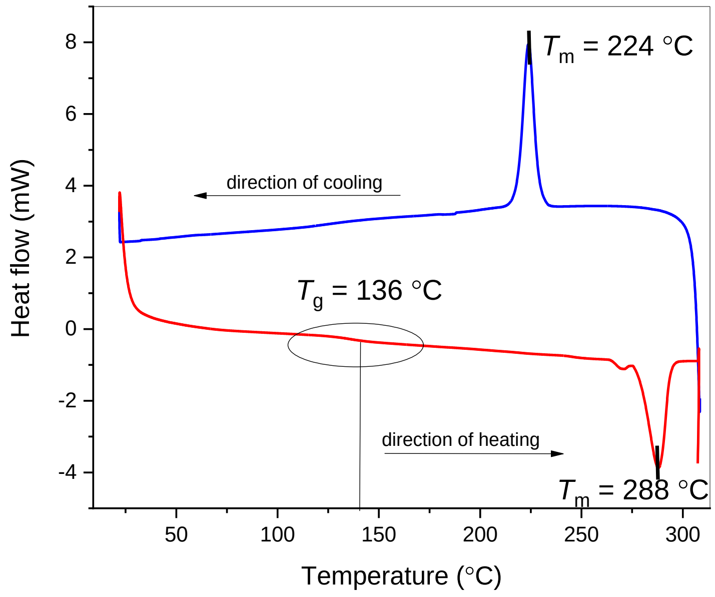

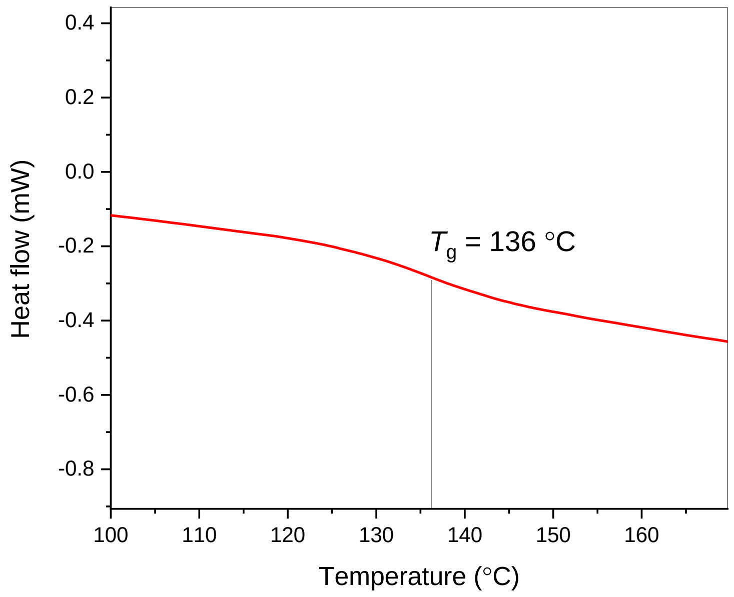

Figure S43. (top) DSC trace collected at 20 °C min^{-1} showing the first cooling and second heating cycle under argon of a 50:50 blend of poly(**D-1**) ($M_{n,SEC}$ = 9200 g mol^{-1}, $Đ_M$ = 1.15; Table 1, entry 5) and poly(**L-1**) ($M_{n,SEC}$ = 9800 g mol^{-1}, $Đ_M$ = 1.19; Table 1, entry 11), formed by evaporation of $CHCl_3$ (T_g = 136 °C; T_m = 288 °C; T_c = 224 °C). (bottom) Zoomed in section to highlight T_g.

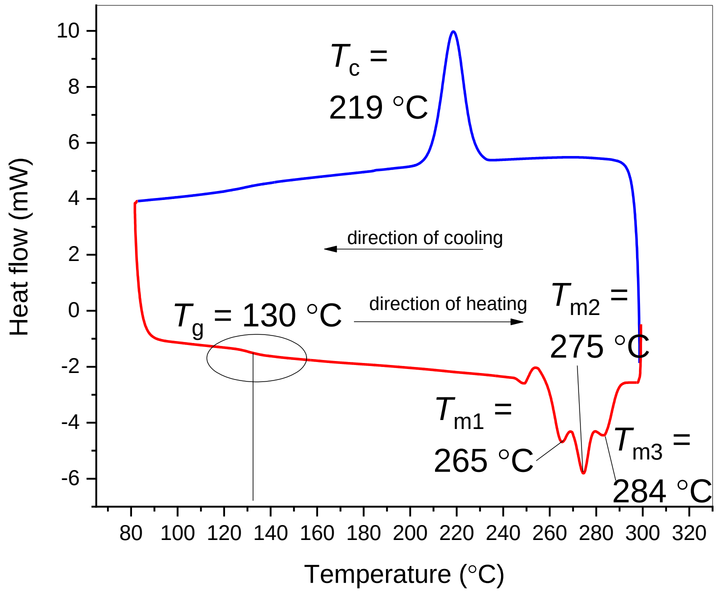

Figure S44. DSC trace collected at 20 °C min^{-1} showing the first cooling and second heating cycle under argon of a 75:25 blend of poly(**L-1**) ($M_{n,SEC}$ = 9800 g mol^{-1}, $Đ_M$ = 1.19; Table 1, entry 11) and poly(**D-1**) ($M_{n,SEC}$ = 9200 g mol^{-1}, $Đ_M$ = 1.15; Table 1, entry 5) formed by evaporation of $CHCl_3$ (T_g = 130 °C; T_{m1} = 264 °C, T_{m2} = 274 °C, T_{m3} = 284 °C; T_c = 219 °C).

10. Wide-angle X-ray scattering (WAXS) analysis

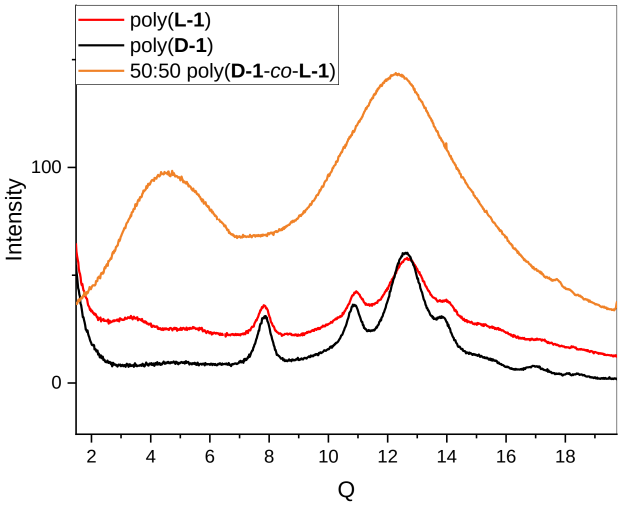

Figure S45. WAXS profiles of poly(**D-1**) (black, $M_{n,SEC}$ = 8300 g mol^{-1}, $Đ_M$ = 1.30), poly(**L-1**) (red, $M_{n,SEC}$ = 9500 g mol^{-1}, $Đ_M$ = 1.18) and 50:50 poly(**D-1**-*co*-**L-1**) (orange, $M_{n,SEC}$ = 9300 g mol^{-1}, $Đ_M$ = 1.17).

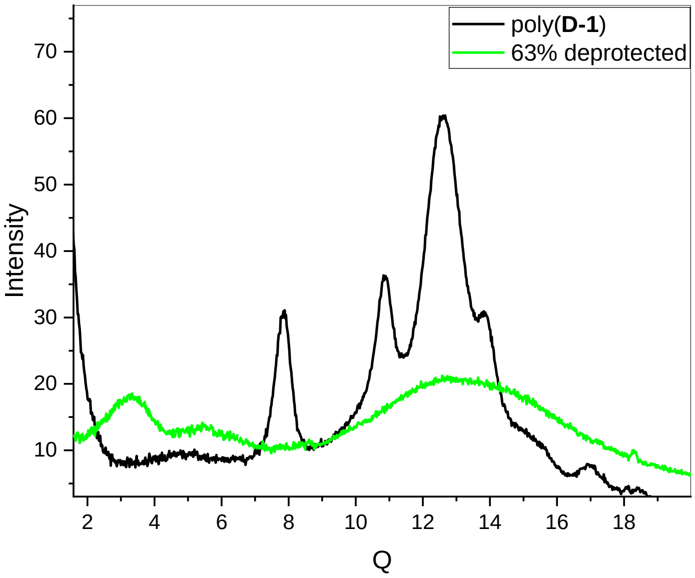

Figure S46. WAXS profiles of poly(**D-1**) (black, $M_{n,SEC}$ = 8300 g mol^{-1}, $Đ_M$ = 1.30) and poly(**D-1**) deprotected at 63% (green).

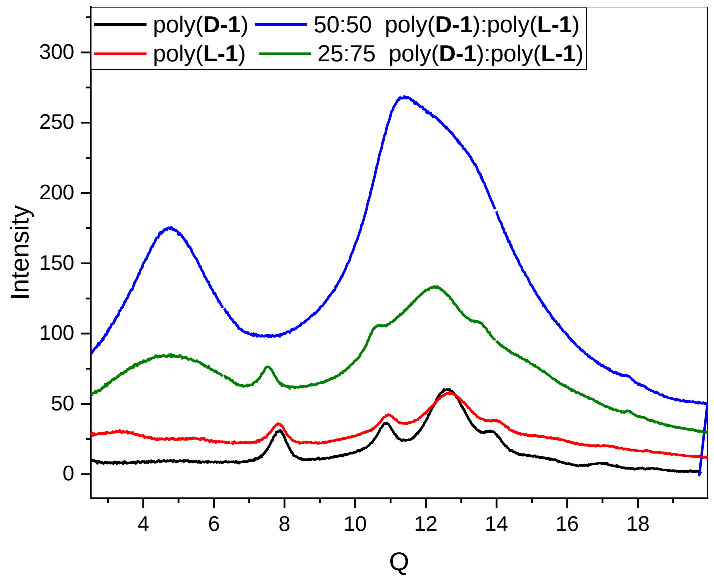

Figure S47. WAXS profiles of poly(**L-1**) (red, $M_{n,SEC}$ = 9400 g mol^{-1}, $Đ_M$ = 1.29), poly(**D-1**) (black, $M_{n,SEC}$ = 9100 g mol^{-1}, $Đ_M$ = 1.25) and 50:50 D:L (blue) and 25:75 D:L (green) blends of poly(**D-1**) ($M_{n,SEC}$ = 9200 g mol^{-1}, $Đ_M$ = 1.15; Table 1, entry 5) and poly(**L-1**) ($M_{n,SEC}$ = 9800 g mol^{-1}, $Đ_M$ = 1.19; Table 1, entry 11).

11. DFT

Density Functional theory (DFT) calculations were performed using Gaussian16 suite of codes (revision A.03).[8] Geometries were fully optimised without any symmetry or geometry constraints. The nature of all the stationary points as minima or transition states (first-order saddle points) on the potential energy surface was verified by calculations of the vibrational frequency spectrum. Free enthalpies were calculated at 423.15 K within the harmonic approximation for vibrational frequencies.

Geometry optimisations were carried out using ωb97XD hybrid functional developed by Chai and Head-Gordon.[9] The 6-311++G(2d,p)[10] basis set was used for O and K atoms and the 6-31G(d,p)[10b, 11] was used for C and H atoms. Solvent effects in tetrahydrofuran were modelled with the SMD[12] continuum model.

All optimised geometries and transition states were subjected to conformational analysis using Schrodinger's Maestro, Macromodel package.[13] A OPLS3e[14] forcefield was applied with no solvent. A Monte Carlo Multiple Minimum (MMCM)[15] /low-mode sampling approach[16] was used to explore the possible conformations of each species. All found conformers were reoptimised with DFT. The lowest energy conformers, once verified as minima or transition states, are included in the free energy diagram (Fig. S43 and Table S3). All DFT-optimised conformers have been uploaded to the Figshare digital repository (see below). Goodvibes software[17] was used to apply temperature and concentration correction factors to calculated free enthalpies at 423.15 K and [**D-1**]$_0$ = 1 mol L^{-1}.

Full coordinates for all the stationary points, together with computed energies and vibrational frequency data, are available *via* the corresponding Gaussian16 output files and calculation spreadsheet, stored in the open-access digital repository DOI 10.6084/m9.figshare.13215617 (https://figshare.com/s/90d8b9d008665fe3c3cd).

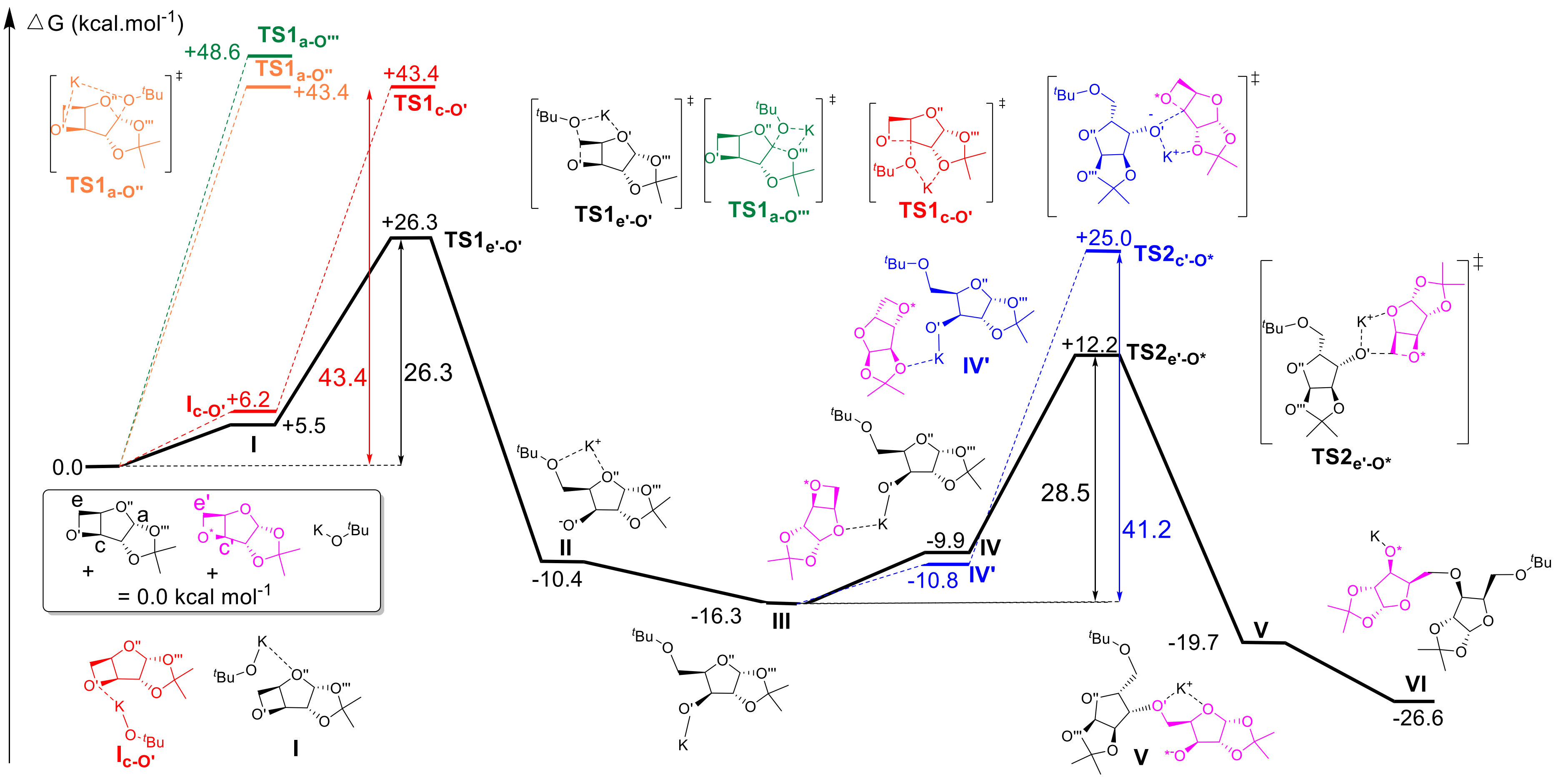

Figure S48. DFT computed profile for the initiation (**TS1**) and propagation (**TS2**) of **D-1**.

Note: Conformational analysis of the transition states revealed that the lowest energy pathways involved K⁺ interacting with the furanose ring, rather than with the oxygen (O') of the oxetane ring.

Table S3. computed Free Gibbs Energy of structures given in Fig. S43 and their ΔG relative to **D-1** and KO^{t}Bu (for initiation step; or 2 × **D-1** and KO^{t}Bu for the propagation step)

Structure	G (Hartree)	ΔG (kcal mol^{-1})
**D-1**	–612.587234	-
**KO^{t}Bu**	–832.895941	-
**D-1 + KO^{t}Bu** (reference for initiation step)	–1445.483175	0.0
**I**	–1445.474332	+5.5
**TS1$_{e\text{-}O'}$**	–1445.432936	+26.3
**II**	–1445.499803	–10.4
**III**	–1445.509130	+16.3
**2 × D-1 + KO^{t}Bu** (reference for propagation step)	–2058.070409	0.0
**IV**	–2058.073111	–9.9
**TS2$_{e'\text{-}O*}$**	–2058.050967	+12.2
**V**	–2058.101759	–19.7
**VI**	–2058.112791	–26.6
**I$_{c\text{-}O'}$**	–1445.47329	+6.2
**TS1$_{c\text{-}O'}$**	–1445.413972	+26.3
**TS1$_{a\text{-}O''}$**	–1445.414041	+43.4
**TS1$_{a\text{-}O'''}$**	–1445.405734	+48.6
**IV'**	–2058.087666	–10.8
**TS2$_{c'\text{-}O*}$**	–2058.030577	+25.0

12. Phosphorylation of deprotected poly(D-1)

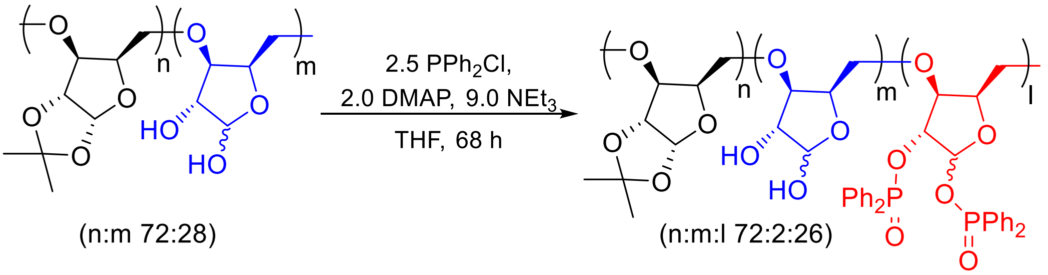

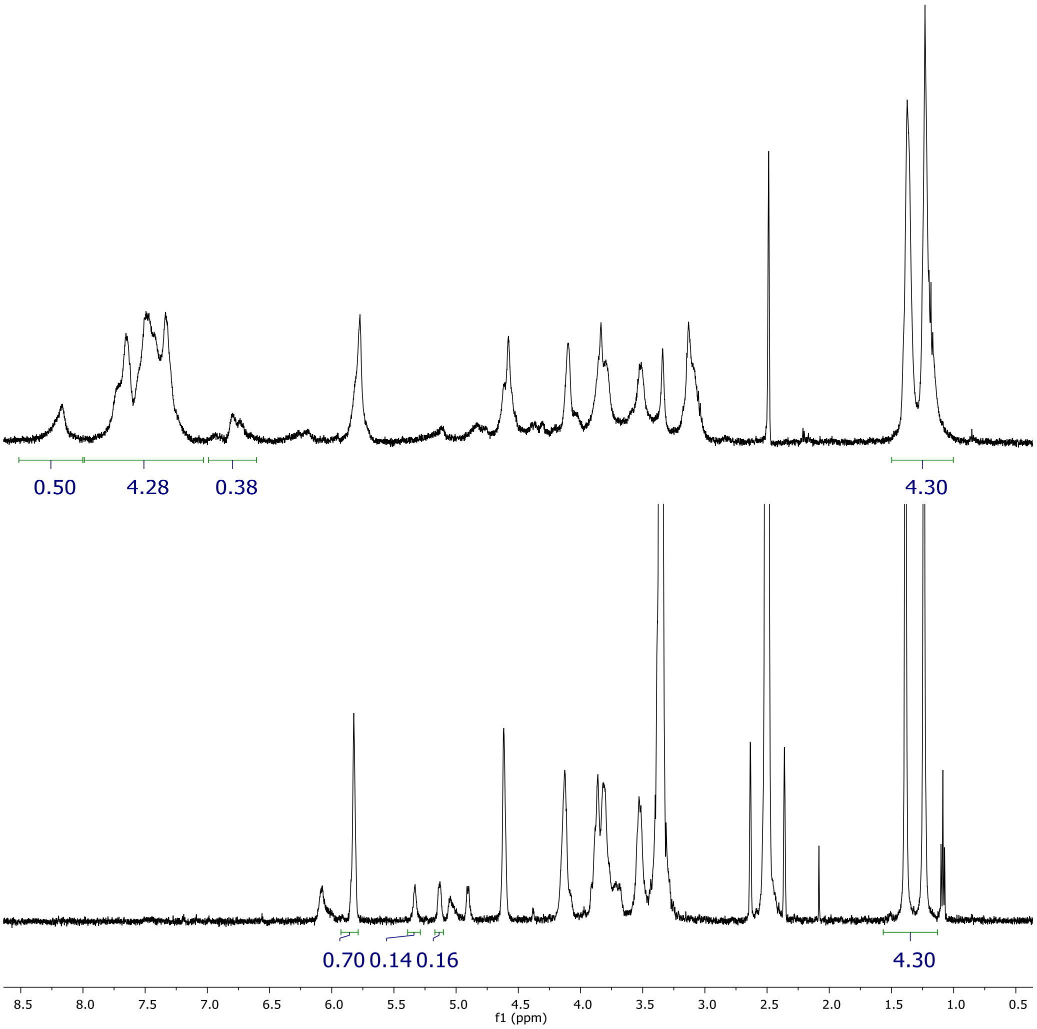

Figure S49. ^{1}H NMR spectra (d^{6}-DMSO) of: (top) isolated polymer phosphorylated at 25% (relative integration of aromatic signals (20 protons) *vs* methyl acetal groups (6 protons)) ($M_{n,SEC}$ = 10100 g mol^{-1}, $Đ_M$ = 1.19); (bottom) parent poly(**D-1**) deprotected at 28% (bottom, $M_{n,SEC}$ = 10700 g mol^{-1}, $Đ_M$ = 1.23).

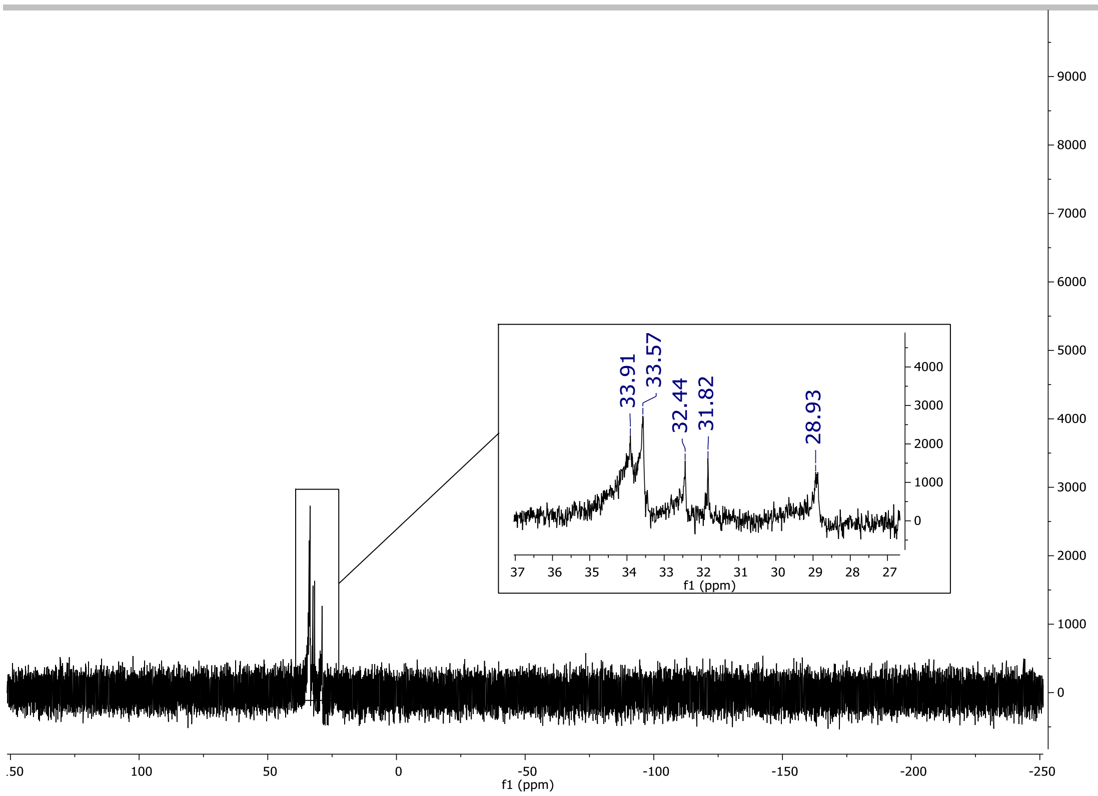

Figure S50. $^{31}P\{^{1}H\}$ NMR spectrum (d^6-DMSO) of poly(**D-1**) phosphorylated at 25% ($M_{n,SEC}$ = 10100 g mol^{-1}, $Đ_M$ = 1.19).

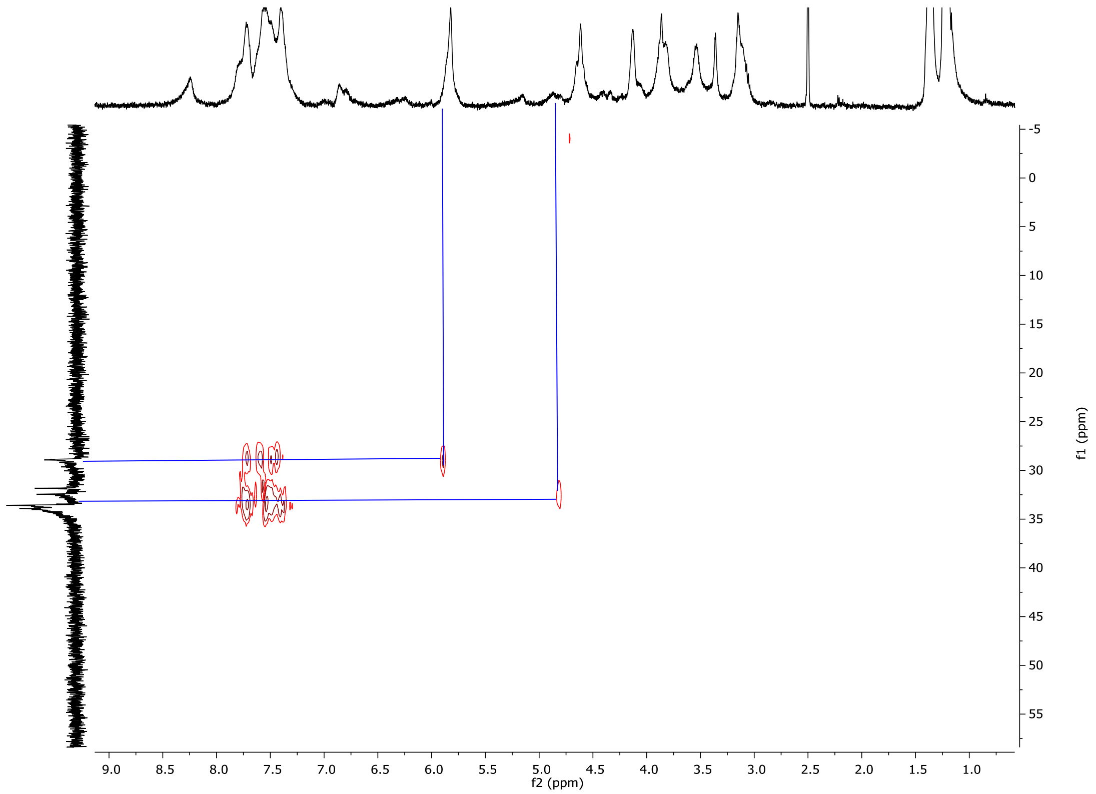

Figure S51. ^{1}H-$^{31}P\{^{1}H\}$ HMBC (d^6-DMSO) spectrum of poly(**D-1**) phosphorylated at 25% ($M_{n,SEC}$ = 10100 g mol^{-1}, $Đ_M$ = 1.19).

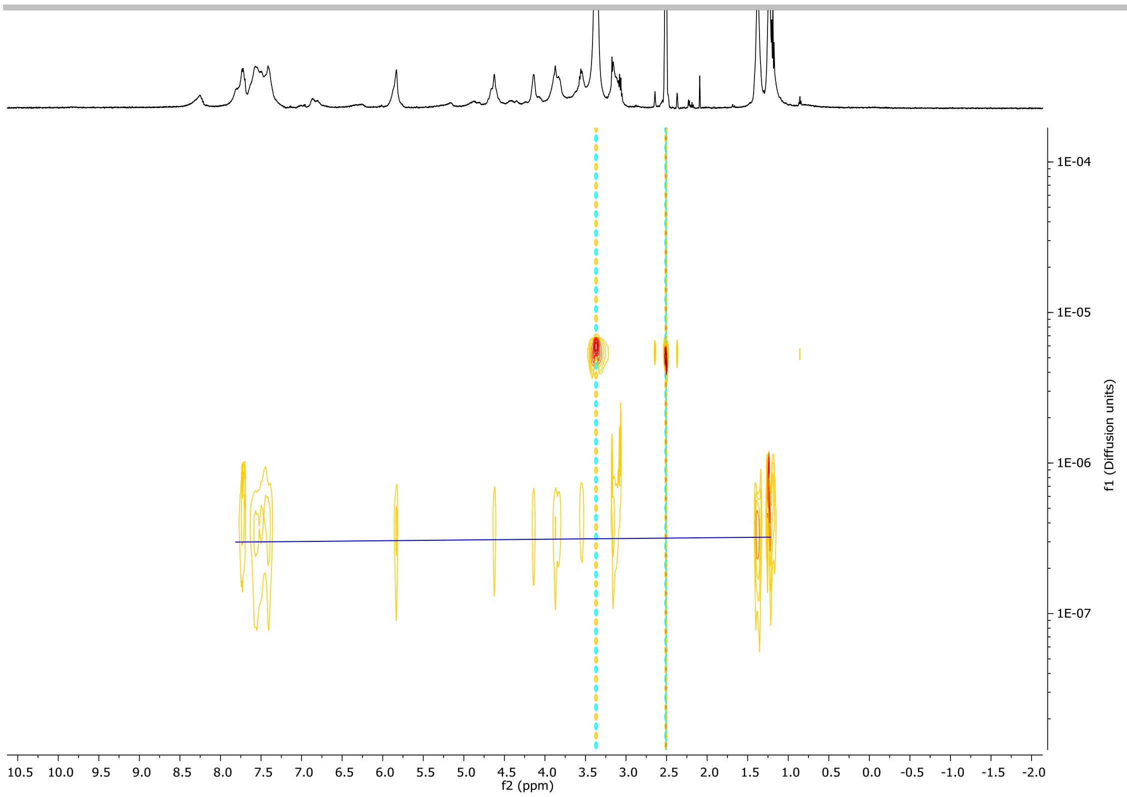

Figure S52. ^{1}H DOSY NMR spectrum (d^6-DMSO) of poly(**D-1**) phosphorylated at 25% ($M_{n,SEC}$ = 10100 g mol^{-1}, $Đ_M$ = 1.19).

13. Optical rotation data

Table S4. Specific rotations of selected compounds (recorded at 25 °C in $CHCl_3$ solutions (concentrations 2–8 mg mL^{-1}))

Entry	Compound	Specific rotation
**1**	**D-Ts-IPXF**	+13.6°
**2**	**L-Ts-IPXF**	–12.0°
3	**D-1**	+13.8
4	**L-1**	–13.2°
5	Poly(**D-1**)	–85.3°
6	Poly(**L-1**)	+85.8°
7	Poly(**D-1-*co*-L-1**)	–0.6°

References

[1] F. da Paixao Soares, E. Groaz, P. Herdewijn, *Molecules* **2018**, *23*, 1457.
[2] E. Paz-Morales, R. Melendres, F. Sartillo-Piscil, *Carbohydr. Res.* **2009**, *344*, 1123-1126.
[3] Y. Q. Shen, X. H. Chen, R. A. Gross, *Macromolecules* **1999**, *32*, 2799-2802.
[4] M. Piccini, D. J. Leak, C. J. Chuck, A. Buchard, *Polym. Chem.* **2020**, *11*, 2681-2691.
[5] Y. X. Lu, G. Just, *Tetrahedron* **2001**, *57*, 1677-1687.
[6] J. Moravcova, P. Rollin, C. Lorin, V. Gardon, J. Capkova, J. Mazac, *J. Carbohydr. Chem.* **1997**, *16*, 113-127.
[7] a) T. Y. Uryu, Koyama, Y. Matsuzaki, K., *Makromol. Chem.* **1984**, *185*, 2099-2107; b) T. Y. Uryu, Koyama, Y. Matsuzaki, K., *J. Polym. Sci. Polym. Lett. Ed.* **1979**, *17*, 673-678.
[8] M. J. Frisch, G. W. Trucks, H. B. Schlegel, G. E. Scuseria, M. A. Robb, J. R. Cheeseman, G. Scalmani, V. Barone, G. A. Petersson, H. Nakatsuji, X. Li, M. Caricato, A. V. Marenich, J. Bloino, B. G. Janesko, R. Gomperts, B. Mennucci, H. P. Hratchian, J. V. Ortiz, A. F. Izmaylov, J. L. Sonnenberg, Williams, F. Ding, F. Lipparini, F. Egidi, J. Goings, B. Peng, A. Petrone, T. Henderson, D. Ranasinghe, V. G. Zakrzewski, J. Gao, N. Rega, G. Zheng, W. Liang, M. Hada, M. Ehara, K. Toyota, R. Fukuda, J. Hasegawa, M. Ishida, T. Nakajima, Y. Honda, O. Kitao, H. Nakai, T. Vreven, K. Throssell, J. A. Montgomery Jr., J. E. Peralta, F. Ogliaro, M. J. Bearpark, J. J. Heyd, E. N. Brothers, K. N. Kudin, V. N. Staroverov, T. A. Keith, R. Kobayashi, J. Normand, K. Raghavachari, A. P. Rendell, J. C. Burant, S. S. Iyengar, J. Tomasi, M. Cossi, J. M. Millam, M. Klene, C. Adamo, R. Cammi, J. W. Ochterski, R. L. Martin, K. Morokuma, O. Farkas, J. B. Foresman, D. J. Fox, Gaussian, Inc., Wallingford, CT, Gaussian 16 Rev. A.03, **2016**.
[9] a) J.-D. Chai, M. Head-Gordon, *Phys. Chem.* **2008**, *10*, 6615-6620; b) J.-D. Chai, M. Head-Gordon, *Chem. Phys. Lett.* **2008**, *467*, 176-178.
[10] a) R. Krishnan, J. S. Binkley, R. Seeger, J. A. Pople, *J. Chem. Phys.* **1980**, *72*, 650-654; b) T. Clark, J. Chandrasekhar, G. W. Spitznagel, P. V. R. Schleyer, *J. Comput. Chem.* **1983**, *4*, 294-301.
[11] W. J. Hehre, R. Ditchfield, J. A. Pople, *J. Chem. Phys.* **1972**, *56*, 2257-2261.
[12] A. V. Marenich, C. J. Cramer, D. G. Truhlar, *J. Phys. Chem. B* **2009**, *113*, 6378-6396.
[13] a) F. Mohamadi, N. G. J. Richards, W. C. Guida, R. Liskamp, M. Lipton, C. Caufield, G. Chang, T. Hendrickson, W. C. Still, *J. Comput. Chem.* **1990**, *11*, 440-467; b) Schrödinger Release 2019-2: MacroModel, Schrödinger, LLC, New York, NY **2019**.
[14] E. Harder, W. Damm, J. Maple, C. Wu, M. Reboul, J. Y. Xiang, L. Wang, D. Lupyan, M. K. Dahlgren, J. L. Knight, J. W. Kaus, D. S. Cerutti, G. Krilov, W. L. Jorgensen, R. Abel, R. A. Friesner, *J. Chem.* **2016**, *12*, 281-296.
[15] G. Chang, W. C. Guida, W. C. Still, *J. Am. Chem. Soc.* **1989**, *111*, 4379-4386.
[16] I. Kolossváry, W. C. Guida, *J. Comput. Chem.* **1999**, *20*, 1671-1684.
[17] a) G. Luchini, J. V. Alegre-Requena, I. Funes-Ardoiz, R. S. Paton, *F1000Research* **2020**, *9*, 291; b) I. Funes-Ardoiz, R. S. Paton, **2018**, *doi:10.5281/zenodo.595246*.

Author Contributions

A. B. and T. M. G. conceived the project. T. M. G. and E. D. designed and performed the synthetic experiments, supervised by A. B. T. M. G., J. B. and E. H. F. carried out computational modelling, supervised by A. B. and M. N. G. All authors prepared the manuscript.
